# Eliciting Dual-Niche Immunological Priming by Acupoint Delivery of Nanovaccines

**DOI:** 10.1007/s40820-025-01789-y

**Published:** 2025-05-28

**Authors:** Lu Wang, Yanhong Sun, Meiling Yan, Lihua Wang, Yiyang Wang, Mengmeng Zhang, Qian Li, Huangan Wu, Jinyao Liu, Chunhai Fan

**Affiliations:** 1 Shanghai Key Laboratory for Nucleic Acid Chemistry and Nanomedicine, Institute of Molecular Medicine, State Key Laboratory of Systems Medicine for Cancer, Shanghai Cancer Institute, Renji Hospital, School of Medicine, Shanghai Jiao Tong University, Shanghai, 200127 People’s Republic of China; 2 Institute of Materiobiology, Department of Chemistry, College of Science, Shanghai University, Shanghai, 200444 People’s Republic of China; 3 Division of Physical Biology and Bioimaging Center, Shanghai Synchrotron Radiation Facility, CAS Key Laboratory of Interfacial Physics and Technology, Shanghai Institute of Applied Physics, Chinese Academy of Sciences, Shanghai, 201800 People’s Republic of China; 4 School of Chemistry and Chemical Engineering, Frontiers Science Center for Transformative Molecules and National Center for Translational Medicine, Shanghai Jiao Tong University, Shanghai, 200240 People’s Republic of China; 5 Key Laboratory of Acupuncture and Immunological Effects, Shanghai University of Traditional Chinese Medicine, Shanghai, 200030 People’s Republic of China; 6 Jiaxing Key Laboratory of Biosemiconductors (A), Xiangfu Laboratory, Jiashan, 314102 People’s Republic of China

**Keywords:** Nanovaccines, Acupoint, Drug delivery, Antigen presentation, Immunization

## Abstract

Immunization has long played essential roles in preventing diseases. However, the desire for precision delivery of vaccines to boost a robust immune response remains largely unmet. Here, we describe the use of acupoint delivery of nanovaccines (ADN) to elicit dual-niche immunological priming. ADN can simultaneously stimulate mast cell-assisted maturation of dendritic cells at the acupoint and enable direct delivery of nanovaccines into the draining lymph nodes. We demonstrate that ADN not only provokes antigen presentation by lymph node-resident CD8α^+^ dendritic cells, but also induces the accumulation of nanovaccines in B-cell zones, amplifying antigen-specific cytotoxic T lymphocyte responses and immunoglobulin G antibody expression in draining lymph nodes. ADN also generates systemic immune responses by causing immune memory and preventing T-cell anergy in the spleen. Further supported by evoking effective antitumor responses and high-level antiviral antibodies in mice, ADN provides a simple yet versatile platform for advanced nanovaccination.

**Supplementary Information:**

The online version contains supplementary material available at 10.1007/s40820-025-01789-y.

## Highlights

- We report the use of acupoint delivery of nanovaccines (NVs) to elicit a dual-niche immunological priming by simultaneously stimulating mast cell-assisted maturation of CD11b^+^ dendritic cells (DCs) at the acupoint and directly enriching NVs into the draining lymph nodes (dLNs).
- The dual-niche immunological priming not only provokes antigen presentation by LN-resident CD8α^+^ DCs, but also induces the accumulation of NVs in B-cell zones, highlights a facile yet versatile platform for advanced nanovaccination toward tumor therapy and infection prevention.

**Supplementary Information:**

The online version contains supplementary material available at 10.1007/s40820-025-01789-y.

## Graphical abstract

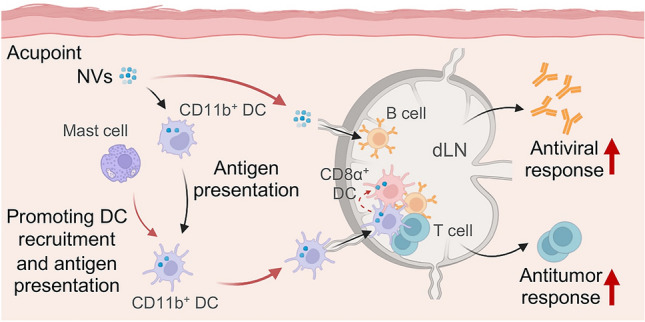

## Introduction

Immunization is an important approach to prevent or even treat diseases by eliciting antigen-specific immune responses [1, 2]. Parenteral administration, particularly intramuscular (IM) injection, is widely applied for immunization [3]. Following injection, tissue-resident antigen-presenting cells (APCs) internalize vaccines and subsequently migrate to draining lymph nodes (dLNs) to induce antigen cross-presentation [4–6]. Simultaneously, a small portion of administered vaccines can directly diffuse into dLNs to elicit immunological priming [7–9]. However, insufficient distribution of APCs in the site of injection inevitably causes either limited transferring of antigens to dLN-resident APCs or inadequate cross-presentation to T cells in dLNs [10]. Meanwhile, the existence of multiple biological barriers at organ and sub-organ levels can filter the majority of administered vaccines, largely impeding their direct accumulation in dLNs [11]. Consequently, intramuscular injection-mediated immunological priming in dLNs often suffers from low vaccination efficacy due to unsatisfied initial infiltration of tissue-resident APCs and subsequent activation of dLN-resident immune cells [3, 12]. To trigger potent adaptive immune responses, sustained-release devices, use of nanovaccines (NVs), and enhancement of targeting ability have been developed to promote the recruitment of tissue-resident immune cells, the cellular uptake of antigens by APCs, and the delivery of vaccines to dLNs [10, 13–15]. Nevertheless, the effectiveness of conventional vaccinations that solely generate single-niche immunological priming either in the injection site or dLNs remains suboptimal. Novel immunization strategies are thus highly desirable, particularly with respect to emerging threats of rapid spread highly lethal infectious diseases [16].

Acupuncture, or acupoint stimulation, has long been used for alleviating various types of diseases, including chronic pain [17], hypertension [18], stroke rehabilitation [19], rheumatoid arthritis [20], diabetes [21], and others. Acupoints are specific body regions locating either on the skin surface or underneath and characterized by denser distributions of microvessels, lymphatic vessels, and nerves than nonacupoint areas [22, 23]. Acupoint stimulation can induce anatomic structural changes, modulate local innate immune responses, and promote the release of bioactive substances, which synergistically reprogram the acupoint microenvironment [24–26]. These unique characteristics endow acupuncture with remarkable ability to tailor systemic homeostasis and improve therapeutic efficacy of different diseases. Given its effectiveness and favorable safety, the World Health Organization has recommended the use of acupuncture to facilitate the treatment of diseases and disorders for 107 medical conditions [27]. More importantly, whereas the specificity of acupoints has been a focus of argument over the year, more recent evidence has accumulated to substantiate it and reveal the underlying mechanism. A very recent study undoubtedly provides neuroanatomical evidence for the specificity of acupoints in driving vagal–adrenal axis, which transfers electrical and biochemical signals to the central nervous system to modulate body physiology [25].

**Fig. 1:**
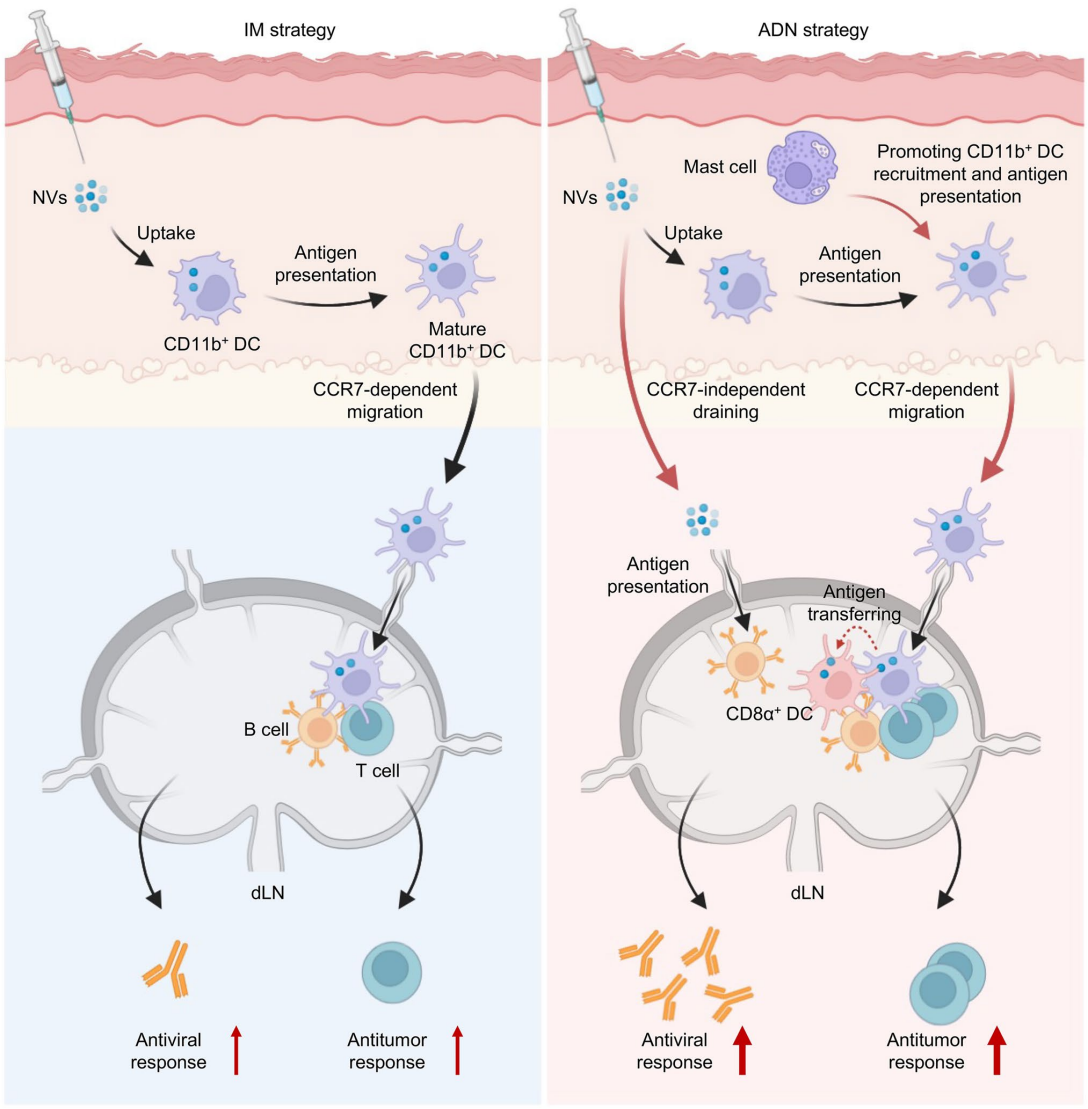
Schematic illustration of AND-mediated elicitation of dual-niche immunological priming. ADN induces enhanced recruitment of tissue-resident CD11b^+^ DCs and antigen presentation at acupoint region in a mast cell-dependent way, and enables direct accumulation of NVs into dLNs in a CCR7-independent manner, boosting robust antitumor and antiviral adaptive immune responses in dLNs. Graphic created with BioRender.com

Here, we report the elicitation of dual-niche immunological priming by acupoint delivery of NVs (ADN) (Fig. 1). Following acupoint injection, NVs induce the upregulation of CD40 ligand (CD40L) expression on acupoint-resident mast cells and enhance their degranulation. Together with concentrated distribution of mast cells at acupoint region, ADN facilitates the recruitment of tissue-resident CD11b^+^ dendritic cells (DCs) and promotes their antigen presentation. On the other hand, ADN enables direct delivery of NVs into dLNs in a CC-chemokine receptor 7 (CCR7)-independent manner. We find that the migration of mature tissue-resident CD11b^+^ DCs into dLNs provokes antigen presentation by dLN-resident CD8α^+^ DCs. Meanwhile, direct exposure of NVs in dLNs induces the accumulation of nanovaccines in B-cell zones, resulting in germinal center (GC) formation. In addition to the amplification of antigen-specific cytotoxic T lymphocyte (CTL) responses and immunoglobulin G (IgG) antibody expression in dLNs, ADN also stimulates systemic immune responses by generating immune memory and preventing T-cell anergy in the spleen. Compared to Houhai acupoint (GV1) and Neiguan acupoint (PC6), ADN at Zusanli acupoint (ST36) consistently triggers the strongest adaptive immune response. Furthermore, we disclose the dose-dependent efficacies of ADN and the difference of vaccination outcomes among unilateral, bilateral, and combined routes of acupoint injection. Utilizing severe acute respiratory syndrome-coronavirus 2 (SARS-CoV-2) spike 1 (S1) protein and ovalbumin (OVA) as model antigens, ADN achieves robust production of anti-S1 subunit IgG antibody and potently suppresses the growth of tumors and LN metastasis in a murine model of OVA-overexpressed melanoma. This work sheds lights on the significance of acupoints in NV-based immunization and unravels the potential mechanisms underlying immunoactivation. We anticipate that ADN provides a facile yet universal approach to enhance the efficacy of a broad variety of NVs.

## Experimental Section

### Materials

Purified anti-mouse CD16/32 (101,302, clone:93), PerCP-Cy5.5-labeled anti-mouse B220 (103,222, RA3-6B2), Alexa Fluor 594-labeled anti-mouse/human B220 (103,254, RA3-6B2), Alexa Fluor 647-labeled anti-mouse/human GL7 (144,606, GL7), APC-labeled anti-mouse CD138 (142,506, clone: 281–2), FITC-labeled anti-mouse CD69 (104,505, clone: H1.2F3), FITC-labeled anti-mouse CD11c (117,306, clone: N418), PerCP-Cy5.5-labeled anti-mouse CD11b (101,228, clone: M1/70), APC-labeled anti-mouse CD86 (105,012, clone: 24F), APC-Cy7-labeled anti-mouse CD3 (100,222, clone:17A2), PE-labeled anti-mouse CD8 (100,708, clone: 53–6.7), PerCP-Cy5.5-labeled anti-mouse CD8α (100,734, clone: 53–6.7), APC-labeled anti-mouse CD4 (100,411, clone: GK1.5), PE-Cy7-labeled anti-mouse FcεRIα (134,317, clone: MAR-1), FITC-labeled anti-mouse CD117 (c-kit) (105,805, clone: 2B8), PE-labeled anti-mouse CD154 (CD40L) (157,003, clone: SA047C3), PE-Cy7-labeled anti-mouse CD40 (124,621, clone: 3/23), APC-labeled anti-mouse OX40L (108,811, clone: RM134L), FITC-labeled anti-mouse Ki67 (652,410, clone: 16A8), PE-Cy7-labeled anti-mouse IFN-γ (505,826, clone: XMG1.2), PE-labeled anti-mouse I-A/I-E (MHC-II) (107,630, clone: M5/114.15.2), APC-labeled anti-mouse H-2 Kb bound to SIINFEKL (141,606, clone: 25-D1.16), PerCP-Cy5.5-labeled anti-mouse IL-4 (504,124, clone: 11B11), PerCP-Cy5.5-labeled anti-mouse CD49b (103,519, clone: HMα2), FITC-labeled anti-mouse CD206 (141,704, clone: C068C2), PE-labeled anti-mouse F4/80 (123,110, clone: BM8), and PE-Cy7-labeled anti-mouse CD25 (101,916, clone: 3C7) antibodies for immunostaining were purchased from BioLegend. Anti-mouse B220 (14–0452-82, clone: RA3-6B2), PE-labeled anti-mouse CD80 (12–0801-85, clone: 16-10A1), PE-labeled anti-mouse TNF-α (12–7321-82, clone: MP6-XT22), APC-labeled anti-mouse FoxP3 (17–5773-82, clone: FJK-16 s), PE-labeled anti-mouse CD103 (12–1031-82, clone: 2E7), PE-labeled anti-mouse CD62L (12–0621-83, clone: MEL-14), and PerCP-Cy5.5-labeled anti-mouse CD44 (45–0441-82, clone: IM7) antibodies for immunostaining were purchased from eBioscience. Foxp3/Transcription Factor Fixation/Permeabilization Concentrate and Diluent (00–5521-00) for intracellular stanning was purchased from eBioscience. Anti-Tryptase antibody (ab2378, clone: AA1), anti-CD3 antibody (ab11089, clone: CD3-12), anti-CD8 antibody (ab209775, clone: EPR20305), goat anti-rat IgG H&L conjugated with Alexa Fluor 594 (ab150160, polyclonal antibody), goat anti-rabbit IgG H&L conjugated with Alexa Fluor 488 (ab150077, polyclonal antibody), goat anti-mouse IgG H&L conjugated with Alexa Fluor 488 (ab150113, polyclonal antibody), and goat anti-mouse IgG H&L conjugated with HRP (ab6789, polyclonal antibody) were purchased from Abcam. APC-labeled SIINFEKL-MHC I tetramer was provided by the NIH Tetramer Core Facility. *InVivo*MAb anti-mouse c-Kit (BE0293, clone: ACK2), *InVivo*MAb rat IgG2a isotype control (BE0089, clone: 2A3), and *InVivo*MAb rat IgG2b isotype control (BE0090, clone: LTF-2) were purchased from BioXcell. Anti-mouse CCR7 antibody (MAB3477) was purchased from R&D. Recombinant SARS-CoV-2 S1 protein (40,591-V08H) was purchased from Sino Biological. TMB chromogen solution (E661007-0100) was purchased from Sangon Biotech. OVA (A5503) and R848 (SML0196) were purchased from Sigma-Aldrich. FITC-conjugated OVA (SF069) was purchased from Solarbio, and RBITC-conjugated OVA (bs-0283P-RBITC) was purchased from Bioss. Dopamine hydrochloride (71406A) was purchased from Adamas. PEI was purchased from Sigma-Aldrich.

### Cell Lines and Animals

B16-OVA cells were cultured in RPMI 1640 medium (Gibco, USA) containing 10% fetal bovine serum (Gibco, USA), 100 U mL^−1^ penicillin (Hyclone, USA), and 100 μg mL^−1^ streptomycin (Hyclone, USA) at 37 °C in 5% CO_2_.

Specific-pathogen-free female C57BL/6 or BALB/c wild-type mice (6–8 weeks old) were purchased from Jiesijie (Shanghai, China). All animal protocols were conducted according to the guidelines approved by the Institutional Animal Care and Use Committee of Renji Hospital, Shanghai Jiao Tong University School of Medicine (RJ2023-017A).

### Preparation of NVs

PEI/OVA NVs were prepared by mixing OVA and PEI in deionized (DI) water at different weight ratios, including 1:0.25, 1:0.5, 1:1, and 1:2. Subsequently, the mixtures were stirred at room temperature (RT) for 30 min. The obtained PEI/OVA NVs were stored at 4 °C for further use.

For the preparation of S1/R848 NVs, dopamine hydrochloride (2 mg mL^−1^) was dissolved in 10 mM Tris (pH 8.8) and stirred at a speed of 200 r min^−1^ for 2 h at 37 °C. The synthesized PDA-NPs were washed three times with DI water at 8000 g for 10 min and then resuspended in DI water. Subsequently, PDA-NPs (1 mg) were resuspended in a solution (pH 8.8) containing 0.1 mg mL^−1^ dopamine, 1 mg mL^−1^ of S1 protein, and 200 μg mL^−1^ of R848, with rotation for 1 h at RT. The obtained NVs were purified by washing with DI water thrice at 8500 g for 10 min and then resuspended in PBS for further use.

### Characterization of NVs

The size distribution and zeta potential of the resulting nanoparticles were measured by DLS at 25 °C using Nano ZS (Malvern Instrument, UK). RBITC-conjugated OVA protein or FITC-conjugated S1 protein was employed for the fabrication of NVs to detect the incorporation of antigen using flow cytometry CytoFLEX (Beckman Coulter, USA). In DI water, the concentration and fluorescence intensity of FITC-conjugated OVA at 488 nm in 0–0.1 mg ml^−1^ maintained a linear relationship with the regression equation of y = 0.0007 + 3 × 10^−8^x (R^2^ = 0.997). To determine OVA loading efficiency, the absorbance of remaining FITC-conjugated OVA in the supernatant at 488 nm was detected using a Synergy H1 (BioTek, USA). The loading efficiency of OVA was calculated according to the following formulas: Loading efficiency = ((total mass of added OVA—remaining mass of OVA in the supernatant)/ total mass of added OVA) × 100%. The loading efficiency of S1 protein was calculated according to the same method.

### Transcriptome Sequencing and Pathway Analyses

Total RNA was extracted from the tissue using TRIzol® Reagent according to the manufacturer’s protocol. RNA quality (260/280 and 260/230 ratios) was determined by 5300 Bioanalyzer (Agilent Technologies) and quantified using the NanoDrop (ND-2000, Thermo Fisher Scientific). Then, RNA purification, reverse transcription, library construction, and sequencing were performed at Shanghai Majorbio Bio-pharm Biotechnology Co., Ltd. (Shanghai, China) according to the manufacturer’s instructions (Illumina, San Diego, CA). Briefly, messenger RNA was isolated by oligo(dT) beads and then fragmented by fragmentation buffer. Double-stranded complementary DNA (cDNA) was synthesized using a SuperScript double-stranded cDNA synthesis kit (Invitrogen, CA) with random hexamer primers (Illumina). Then, the synthesized cDNA was subjected to sequencing according to Illumina’s library construction protocol. Libraries were size selected for cDNA target fragments of 300 bp on 2% Low Range Ultra Agarose followed by PCR amplified using Phusion DNA polymerase (New England Biolabs) for 15 PCR cycles. After quantified by Qubit 4.0, paired- end RNA-seq sequencing library was sequenced with the NovaSeq 6000 sequencer (2 × 150 bp read length).

To identify differential expression genes (DEGs) between two different samples, the expression level of each transcript was calculated according to the transcripts per million reads (TPM) method. RSEM was used to quantify gene abundances [28]. Essentially, differential expression analysis was performed using the DESeq2 or DEGseq [29, 30]. DEGs with |log_2_ (fold change)|≥ 1 and false discovery rate (FDR) ≤ 0.05 (DESeq2) or FDR ≤ 0.001 (DEGseq) were identified to be significantly different expressed genes. In addition, functional-enrichment analysis including GO and KEGG was performed to identify which DEGs were significantly enriched in GO terms and metabolic pathways at Bonferroni-corrected *P*-value ≤ 0.05 compared with the whole-transcriptome background. GO functional enrichment and KEGG pathway analysis were performed using Goatools and KOBAS, respectively [31].

### In Vivo Immunization Studies

BALB/c mice aged 6–8 weeks (*n* = 6 in each group) were treated with PBS, PEI, or PEI/OVA NVs (OVA = 30 μg per mouse) by IM or ST36 injection. The ST36 is located below 3–4 mm and lateral 1–2 mm of midline of the knee [25, 32]. A laboratory syringe pump (LSP02-1B, Longer Precision Pump Co., Ltd.) was used to control the injection speed. Using 1 mL syringe fitted with disposable scalp needle (0.45 × 13 mm^2^) withdrew 50 μL NVs and injected into acupoint at rate of 50 μL min^−1^ with a syringe pump. The needle was inserted to a depth of 3 mm. To ensure no liquid leakage, the needle was kept for 2 min after injection and then pulled out slowly. Mice were immunized with PEI/OVA NVs on days 0 and 21. Serum samples were collected on day 14, 28, or 35. On day 1 or 35 post-immunization, tissues of injection site and dLNs were harvested for further assay. For mast cell depletion, NV-immunized mice through ST36 were intravenously injected with 500 μg of anti-c-Kit antibody at −12, 0, and 12 h, respectively. Rat IgG2b was used as a control.

To analyze anti-SARS-CoV-2 spike 1 antibody responses, mice were immunized with S1/R848 NVs following three schemes: (1) S1/R848 NVs (S1 = 20 μg per mouse) injected by IM, PC6, GV1, or ST36 route; (2) different doses of S1/R848 NVs, including S1 doses of 10 g, 20 μg, or 50 μg injected by ST36 route; and (3) equivalent S1/R848 NVs (S1 = 20 μg per mouse) injected by unilateral ST36, bilateral ST36, or unilateral PC6 plus ST36 route. PC6 and GV1 were used to observe the immunization effects of S1/R848 NVs by corresponding acupoint injection [33]. On day 35 post-immunization, serum samples were collected for S1-specific IgG detection by ELISA, and dLNs were harvested for further assay.

### Analysis of ADN-Induced Immune Responses

#### Flow Cytometry Analysis

To analyze the distribution of NVs in dLNs, inguinal dLNs were collected from euthanized mice and then prepared into a single-cell suspension by filtering through a 70-μm cell strainer. To analyze the distribution of NVs among T cells, B cells, DCs, monocytes, and barrier cells (including MCMs, MSMs, and SSMs), the obtained single-cell suspensions were incubated with anti-CD16/32 for 30 min on ice and subsequently stained with PE-labeled anti-CD3, PerCP-Cy5.5-labeled anti-CD45, PE-Cy7-labeled anti-B220, APC-labeled anti-CD11b (CD40L), and APC-Cy7-labeled anti-CD11c. To analyze the abundances or responses of diverse immune cells at the injection sites, the tissues from the injection sites were collected from euthanized mice. Subsequently, the tissues were weighed and cut into pieces. The pieces were incubated in digestion solution (RPMI 1640 containing 10% FBS, 1.5 mg mL^−1^ collagenase IV, and 0.2 mg mL^−1^ DNase I) at 37 °C for 1 h and then filtered through a 70-μm cell strainer to obtain single-cell suspensions. To analyze mast cell activation, the obtained single-cell suspensions were incubated with anti-CD16/32 for 30 min on ice and subsequently stained with PE-Cy7-labeled anti-FcεRIα, FITC-labeled anti-CD117 (c-kit), PE-labeled anti-CD154 (CD40L), or PE-labeled anti-I-A/I-E (MHC-II). To analyze DC activation, single-cell suspensions were incubated with anti-CD16/32 for 30 min on ice and subsequently stained with FITC-labeled anti-CD11c, PerCP-Cy5.5-labeled anti-CD11b, PE-labeled anti-CD103, PE-Cy7-labeled anti-CD40, APC-labeled anti-SIINFEKL/H-2 Kb, PE-labeled anti-CD80, PE-Cy7-labeled anti-CD86, or PerCP-Cy5.5-labeled anti-CD8α. To analyze the immune responses in dLNs, the inguinal dLNs were collected from euthanized mice and then prepared into a single-cell suspension by filtering through the 70-μm cell strainer. To analyze T-cell responses, cells were incubated with purified anti-CD16/32 for 30 min on ice and subsequently stained with APC-Cy7-labeled anti-CD3, APC-labeled anti-CD4, PE-labeled anti-CD8, PerCP-Cy5.5-labeled anti-CD44, PE-labeled anti-CD62L, PE-Cy7-labeled anti-CD25, PE-Cy7-labeled anti-CD40, or APC-labeled SIINFEKL-MHC I tetramer for 45 min on ice. The intracellular proteins were further stained by FITC-labeled anti-Ki67, PE-Cy7-labeled anti-IFN-γ, PerCP-Cy5.5-labeled anti-IL-4, or APC-labeled anti-FoxP3 for 1 h on ice after fixation/permeabilization using Foxp3/Transcription Factor Fixation/Permeabilization Concentrate and Diluent. To analyze B-cell responses, cells were incubated with purified anti-CD16/32 for 30 min on ice and subsequently stained with PerCP-Cy5.5-labeled anti-B220, APC-labeled anti-CD138, and FITC-labeled anti-CD69 for 45 min on ice. To analyze NK cell activation, cells were incubated with purified anti-CD16/32 for 30 min on ice and subsequently stained with APC-Cy7-labeled anti-CD3 and PerCP-Cy5.5-labeled anti-CD49b for 45 min on ice. The intracellular protein was further stained by PE-Cy7-labeled anti-IFN-γ for 1 h on ice after fixation/permeabilization using Foxp3/Transcription Factor Fixation/Permeabilization Concentrate and Diluent. To analyze macrophage polarization, cells were incubated with purified anti-CD16/32 for 30 min on ice and subsequently stained with PE-labeled anti-F4/80, PerCP-Cy5.5-labeled anti-CD11b and PE-Cy7-labeled anti-CD86 for 45 min on ice. The intracellular protein was further stained by FITC-labeled anti-CD206 for 1 h on ice after fixation/permeabilization using Foxp3/Transcription Factor Fixation/Permeabilization Concentrate and Diluent. Flow data were acquired on a FACSVerse flow cytometer (BD Biosciences, USA) and analyzed by FlowJo software (TreeStar).

#### In Vitro Asse﻿ssment of T-Cell Anergy

Splenocytes from mice treated without or with PEI/OVA NVs (OVA = 30 μg per mouse) by IM or ST36 were rechallenged by PEI/OVA NVs (OVA = 10 μg mL^−1^) in 96-well, U-bottom plates for 72 h at 37 °C. Then, splenocytes were collected to detect T-cell proliferation and activation using flow cytometry, which were determined by labeling splenocytes with APC-Cy7-labeled anti-CD3, FITC-labeled anti-Ki67, and PE-labeled anti-TNF-α.

#### ELISA Analysis

To analyze the levels of cytokine, IL-4, IFN-γ, and IFN-β in the serum from treated mice were determined using ELISA kits (Multi Sciences, China) following standard protocols and then quantified on a Synergy H1. The level of OVA- or S1-specific IgG in the serum was also measured by ELISA according to previous description [34]. In brief, the 96-well flat ELISA plates (NEST Biotechnology, China) were coated with 2 μg mL^−1^ recombinant OVA or SARS-CoV-2 S1 protein in carbonate–bicarbonate buffer (pH 9.6) overnight at 4 °C. Plates were blocked with 3% BSA in PBS for 2 h at RT. Next, plates were incubated with serial dilutions of sera in dilution buffer (0.5% BSA in PBS). After incubation for 1 h at 37 °C, plates were incubated with HRP-conjugated goat anti-mouse IgG for 1 h at RT. Washing three times with PBST buffer (PBS buffer containing 0.5% Tween-20) was required between each of the above steps. Finally, the reaction was developed by 3,3’,5,5’-tetramethylbenzidine (TMB) substrate solution and stopped with stop solution (1 M H_2_SO_4_). Plates were determined at 450 nm wavelength within 30 min by a Synergy H1. The endpoint titers were defined as the highest serum dilution, which had an absorbance ≥ twofold over the background values.

#### Immunohistochemistry

To analyze immune activation at the injection sites, tissues from the injection site were removed and fixed with 10% formaldehyde solution. Then, the tissues were dehydrated by gradient ethanol (70%, 80%, 90%, and 100%), cleared with xylene and embedded in paraffin, and cut into 5-μm sections by paraffin microtome (Lacia RM2235). To analyze mast cell degranulation, tissue sections were stained with neutral red dye. To analyze the colocalization of mast cells and DCs, tissue sections were stained with mouse anti-mouse Tryptase antibody followed by incubation with secondary anti-mouse Alexa Fluor 594 antibody and stained with FITC-labeled anti-CD11c.

To observe the immune activation in dLNs, inguinal dLNs from mice were fixed with 4% PFA for 6 h and dehydrated in sucrose solution (10%, 20%, and 30%) at 4 °C, respectively. Sections (10 µm) were prepared by the freezing microtome (LEICA CM1950). Sections of dLNs were incubated with 0.25% Triton X-100 in 5% bovine serum albumin (BSA) for 1 h at RT after washing with PBS for three times. Next, the sections were incubated with APC-labeled anti-CD3, Alexa Fluor 594-labeled anti-B220, or FITC-labeled anti-CD11c for 24 h at 4 °C. Finally, sections were counterstained with 4’,6-diamidino-2-phenylindole (DAPI) for 15 min at RT. Images were acquired using CLSM TCS SP8. The CD11c-positive regions were analyzed by ImageJ software.

To analyze tumor-infiltrating CD8^+^ T cells, the tumor tissues collected at the end of experiment were fixed with 10% formaldehyde solution and embedded in paraffin. The samples were then cut into sections, which were stained with rat anti-mouse CD3 monoclonal antibody and rabbit anti-mouse CD8 monoclonal antibody followed by incubation with secondary anti-rat Alexa Fluor 594 antibody and anti-rabbit Alexa Fluor 488 antibody. Finally, sections were counterstained with DAPI. Images of resultant sample were acquired using CLSM TCS SP8.

### IVIS Imaging

For 3D IVIS imaging, Cy5.5-labeled NVs (OVA = 30 μg per mouse) were injected in volume of 50 μL by IM or ST36 route. On day 3 post-injection, mice were imaged by IVIS Spectrum (Perkin Elmer, USA), and data processing was performed using Living Image® 4.3.2 software. For dLN imaging, mice injected with Cy5.5-, RhB-, or FITC-labeled NVs were euthanized, and dLNs were collected at 6, 24, or 48 h after injection. Fluorescent images of dLNs were acquired with IVIS® Lumina III (Perkin Elmer, USA). To abolish the migration of immune cells, NV-immunized mice through ST36 were intravenously injected with 50 μg of anti-CCR7 antibody on days -1, 0, and 1, respectively. Rat IgG2a was used as a control.

### Tumor Prophylactic and Therapeutic Studies

For the prophylactic study, 6-week-old female C57BL/6 mice were injected with PBS or PEI/OVA NVs (OVA = 30 μg per mouse) by ST36 route on days 0, 5, and 10. On 5 days after the final immunization, treated mice were subcutaneously challenged with 1 × 10^6^ B16-OVA cells. For the therapeutic study, 6-week-old female C57BL/6 mice were subcutaneously injected with 1 × 10^6^ B16-OVA cells. After 7 days inoculation, tumor-bearing mice were immunized with PEI/OVA NVs (OVA = 30 μg per mouse) by IM or ST36 route, or with PEI (30 μg per mouse) every 7 days. The body weights of mice were monitored every other day. The tumor volume was estimated according to the following formula of 0.5 × length × (width)^2^. Mice were recognized as dead after tumor volume reaching 2000 mm^3^. On 17 days post-inoculation, dLNs were collected for analyzing LN metastasis and immune tolerance. Tumor tissues were collected for evaluating immune infiltration.

### Histologic Analysis

Hearts, livers, spleens, lungs, kidneys, dLNs, and tumors of the mice were collected at the end of experiments and fixed with 10% formaldehyde solution. The tissues were then embedded in paraffin and cut into sections for H&E staining. The slides were imaged using NanoZoomer S360 (Hamamatsu).

### Statistical Analysis

All the values are presented as the mean ± s.e.m. with the indicated sample size. All the statistical analyses were carried out with GraphPad Prism software (PRISM 8.0). Data were analyzed by one-way analysis of variance (ANOVA) with the Fisher’s LSD comparison post-test or two-way ANOVA with the Fisher’s LSD post-test for multiple comparisons, and Student’s *t*-test for two-group comparisons. Survival curves were obtained using a log-rank (Mantel–Cox) test. Outliers were identified and removed using the ROUT test (Q = 1%). For all the experiments, significance was defined as a *P* value < 0.05.

## Results and Discussion

### Formation of an Immunological Niche at Acupoints

**Fig. 2:**
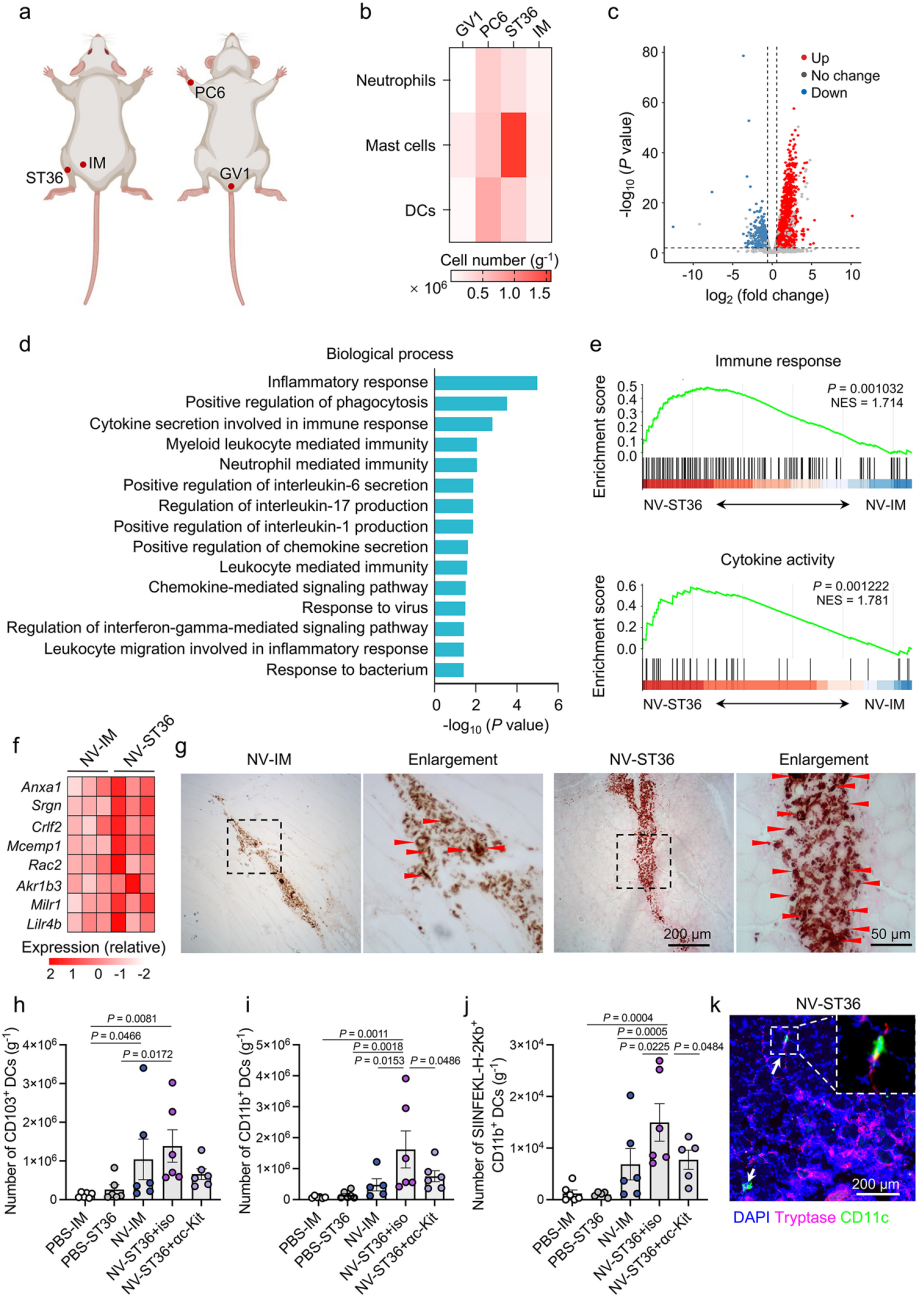
ADN-mediated formation of an immunological niche at ST36.** a** Schematic illustration of the locations of IM, GV1, PC6, and ST36 in mice. **b** Numbers of neutrophils, mast cells, and DCs locating at IM, GV1, PC6, and ST36, respectively (*n* = 4 or 8). **c**-**f** Transcriptomic analysis of tissue samples of the injection sites from mice 6 h post-immunization with PEI/OVA NVs through IM or ST36 route (*n* = 3). **c** Volcano plot showing differentially expressed genes in the NV-ST36 group compared to the NV-IM group. **d** GO enrichment analysis of cluster related to immune functions that were upregulated in the NV-ST36 group compared to the NV-IM group (*P* < 0.05). **e** GSEA showing enrichment of genes of immune response (top) and cytokine activity (bottom) pathways in RNA-seq analysis. NES represents normalized enrichment score. **f** Heatmap representation of the relative expression levels of upregulated genes related to mast cell activation in NV-ST36 and NV-IM groups. **g** Representative images of neutral red-stained tissue sections from the injection site. Red arrows represent granules. Enlarged images were derived from the white dotted squares. **h**-**j** Mice were immunized with PBS or NVs through IM or ST36 route. Mice immunized with NVs through ST36 were intravenously injected with c-kit antibody or isotype control. Tissues at the injection sites were collected at 24 h post-immunization to detect immune responses. Numbers of **h** CD103^+^ DCs, **i** CD11b^+^ DCs, and **j** SIINFEKL^+^ antigen-presenting CD11b^+^ DCs at the injection site. **k** Representative immunofluorescence images of ST36 tissue sections stained with anti-Tryptase antibody (magenta), anti-CD11c antibody (green), and 4’,6-diamidino-2-phenylindole (DAPI, blue) on day 3 post-immunization. White arrows represent the contact between mast cells and DCs. The data are presented as the mean ± s.e.m. Statistical analysis was performed by one-way ANOVA with Fisher’s LSD post-test

Given that the quantity and phenotypes of tissue-resident immune cells at the injection site profoundly influence the antigen presentation by APCs [10], we separately assessed the immune cell profiling in IM, GV1, PC6, and ST36 in the steady state. The location and depth of these acupoints were determined according to International Standard Acupuncture Points of World Health Organization and the transpositional animal acupoint system for mouse model (Fig. 2a). GV1 is located in the depression between the anus and the tail base; PC6 is located in the medial side of the forelimb and 3 mm below the wrist, between the ulnar and radial joints; ST36 is located below 3–4 mm and lateral 1–2 mm of midline of the knee. IM is located in the middle of femur and tibia and 3 mm below the epidermis. As evidenced in Fig. 2b, mast cells were more abundant at acupoint sites, especially ST36, compared to IM region. It has been reported that mast cells are crucial initiators and effectors of innate immunity, which can further facilitate the magnitude and duration of adaptive immune responses [35]. We thus hypothesized that acupoints could offer a unique immunoactive microenvironment for NVs. As a proof-of-concept study, we chose polyethyleneimine (PEI) as the nanocarrier material due to its extensive application in protein and nucleotide delivery [36]. Note that PEI-based NVs with optimized safety have been applied in patients with pancreatic adenocarcinoma (NCT01274455) or neuroblastoma (NCT04049864). In addition, OVA was selected as model antigen because it is a typical T-cell-dependent antigen for studying antigen-specific adaptive immunity in mice [37]. To form NVs by nanocomplexing, PEI and OVA were mixed under vibration at different weight ratios (Fig. S1a). Dynamic light scattering (DLS) measurement suggested that the obtained nanoparticles displayed hydrodynamic sizes in the range of 110–200 nm (Fig. S1b), with comparable surface zeta potentials around + 38 mV (Fig. S1c). PEI/OVA NVs (PEI:OVA = 1:1 w/w) with an average size of ~ 110 nm were applied as a relatively small size can facilitate cell uptake [38]. The loading of antigen was separately evidenced by confocal laser scanning microscopy (CLSM) and flow cytometric analysis, showing a sharp increment in fluorescence intensity of NVs loaded with OVA-RhB (Fig. S1d, e). The antigen loading content was calculated to be 62 wt% by optimizing feeding concentration of OVA (Fig. S1f).

To panoramically characterize the potency of ADN, transcriptomic analysis of tissue samples of IM (termed as NV-IM) and ST36 (termed as NV-ST36) sites was conducted at 6 h post-immunization. RNA sequencing data demonstrated the differentially expressed gene transcripts between IM and ST36 routes, among which 2289 genes were upregulated and 1500 genes were downregulated in the tissue samples of the NV-ST36 group compared to those of the NV-IM group (Fig. 2c). The Kyoto Encyclopedia of Genes and Genomes (KEGG) enrichment analysis revealed that the most significantly enriched pathways were mainly associated with ribosome, oxidative phosphorylation, and thermogenesis (Fig. S2a), which participate in several homeostatic functions associated with host defense. Particularly, Gene Ontology (GO) and Gene-set enrichment analysis (GSEA) further disclosed the upregulated immune-related pathways including immune response, cytokine activity, and chemokine activity in the NV-ST36 group (Figs. 2d, e and S2b). Correspondingly, a number of interferon genes were upregulated in the NV-ST36 group (Fig. S2c). In comparison with IM injection, ADN demonstrated a significant increment in activated DCs at the injection site (Fig. S2d). Note that ADN induced the increased expressions of several genes related to mast cell activation (Anxa1, Srgn, Crlf2, Mcemp1, Rac2, Akr1b3, Milr1, and Lilr4b) (Fig. 2f). The promoted activation of mast cells was further confirmed by mast cell granules using neutral red staining of the muscle tissues (Fig. 2g), displaying more secretory granules that contain preformed bioactive substances, such as amines or cytokines, to tailor the immunoactive milieu [39, 40].

To confirm whether mast cell accumulation and activation could promote the recruitment of immune cells to the injection site, tissue-resident DCs, which migrate from the injection site to dLNs after activation [5], were detected using flow cytometric analysis. Phosphate-buffered saline (PBS) injected to IM (termed PBS-IM) or ST36 region (termed PBS-ST36) was carried out as controls. As shown in Fig. 2h, immunization largely recruited migratory CD103^+^ DCs to the injection site, yet showing comparable levels of CD103^+^ DCs between the NV-IM and NV-ST36 groups. Of note, only ST36 injection exhibited remarkable recruitment of migratory CD11b^+^ DCs (Fig. 2i), which play a crucial role in T-cell priming and are responsible for T-cell-dependent antibody responses [4]. In line with the notion that the C–C motif chemokine ligand 9 (CCL9) is pivotal for the recruitment of CD11b^+^ DCs [41], transcriptomic analysis confirmed a significantly increased expression of CCL9 in the NV-ST36 group (Fig. S2e). We then examined the presentation by CD11b^+^ DCs in immunized mice. As expected, ST36 injection resulted in the highest level of SIINFEKL-H-2 Kb^+^CD11b^+^ DCs (Fig. 2j), which disclosed more effective in facilitating antigen presentation. Worth noting, the enhanced recruitment of CD11b^+^ DCs and antigen presentation by CD11b^+^ DCs in the NV-ST36 group were greatly suppressed by mast cell depletion (Fig. 2i, j), affirming the indispensable role of mast cells in promoting ADN-mediated priming. Immunofluorescence staining of mast cells and DCs from immunized mice via ST36 route further demonstrated the closely direct contact between mast cells and DCs (Figs. 2k and S3), which is reported to facilitate antigen transfer for T-cell activation [42].

Given that costimulatory molecules play a critical role in mast cell-DC interactions [43], the levels of OX40 ligand (OX40L), major histocompatibility complex class (MHC)-II, and CD40L on mast cells were detected. Different from unchanged expression of OX40L (Fig. S4a), the level of MHC-II on mast cells was upregulated in mice treated with NVs (Fig. S4b). More importantly, only ST36 injection evoked the expression of CD40L on mast cells (Fig. S4c), and correspondingly increased the expression of CD40 on DCs (Fig. S4d). These results indicated that CD40-CD40L interaction might be involved in the mast cell-mediated promotion of presentation by tissue-resident CD11b^+^ DCs. In addition, the mast cell stabilizer of disodium cromoglycate (DSCG), that can diminish mast cell activation [44], was also used to verify the dependency of immunological priming on mast cells. As shown in Fig. S4b-d, treatment with DSCG blunted the upregulations of MHC-II and CD40L on mast cells, and correspondingly reduced the expression of CD40 on DCs induced by ADN. DSCG-treated mice also showed dramatically inhibited recruitment of CD11b^+^ DCs and presentation by CD11b^+^ DCs (Fig. S4e-g). As predicted, ADN-triggered direct contact between mast cells and DCs was blocked after treatment with DSCG (Fig. S3). Overall, these results disclosed that ADN could form a local mast cell-activated niche to amplify NV-triggered DC maturation.

### Formation of an Immunological Niche in dLNs

**Fig. 3:**
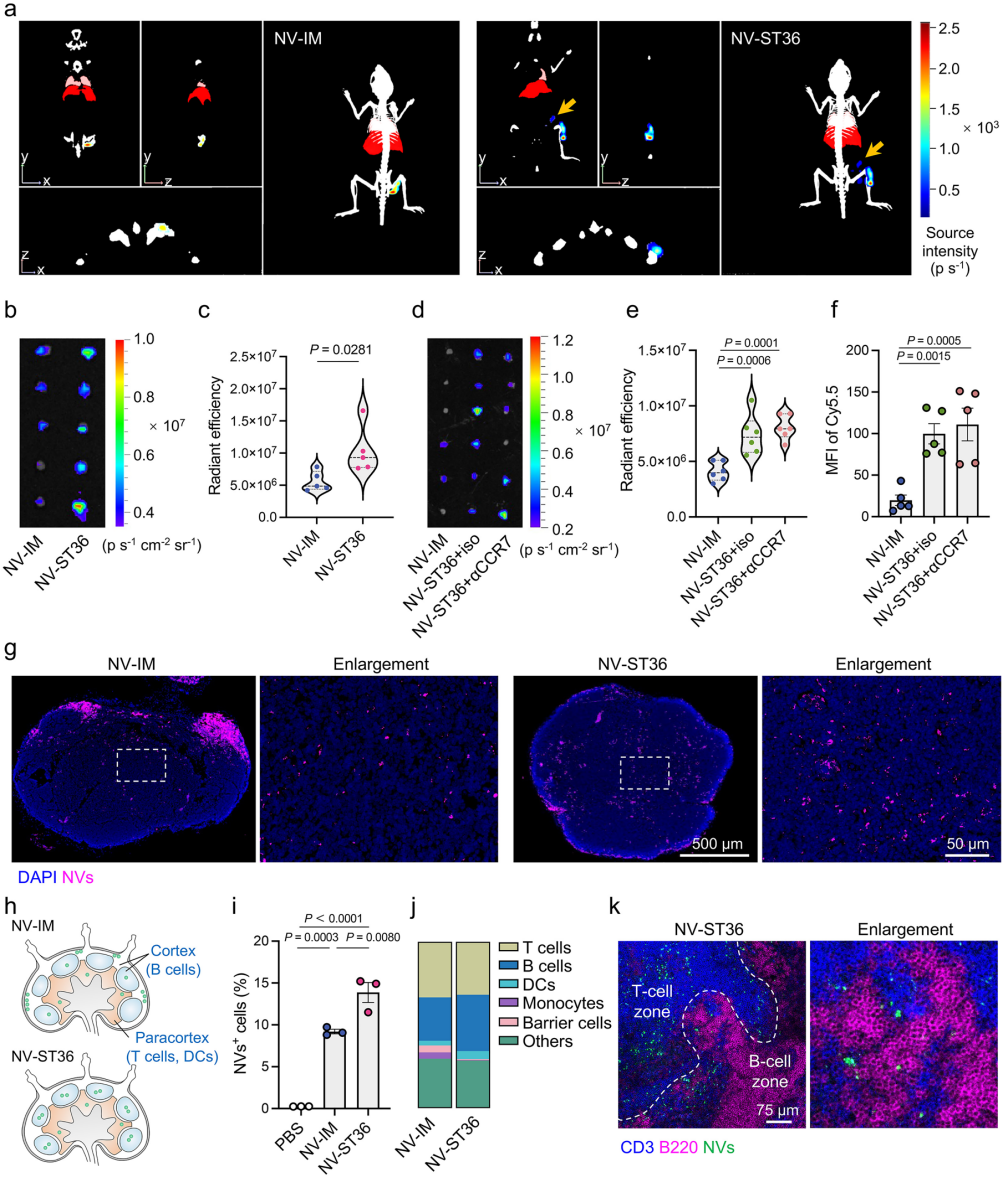
ADN-mediated formation of an immunological niche in dLNs. **a** Representative 3D IVIS images of mice 3 days post-immunization with Cy5.5-labeled NVs through IM or ST36 route. Yellow arrows represent dLNs. **b**-**f** Mice were injected with Cy5.5- or FITC-labeled NVs through IM or ST36 route. Mice immunized through ST36 were intravenously injected with CCR7 antibody or isotype control. The dLNs were collected at 48 h post-immunization. **b**, **d** Representative IVIS images and **c**, **e** corresponding fluorescence intensities of the sampled dLNs (*n* = 5 or 6).** f** MFI of Cy5.5 of single LN cell suspensions measured by flow cytometry (*n* = 5). **g** Representative CLSM images of dLN tissues sectioned from mice 7 days post-immunization. Magenta and blue indicate NVs and DAPI-labeled nuclei, respectively. Enlarged images were derived from the white dotted squares. **h** Schematic illustration of the distribution of NVs in dLNs. **i** Percentage of NV-positive cells in dLNs measured by flow cytometry (*n* = 3). **j** Proportions of T cells, B cells, DCs, monocytes, and barrier cells (MCMs, MSMs, and SSMs) among NV-positive cells in dLNs (*n* = 3). **k** Representative immunofluorescence images of dLN sections stained with anti-CD3 antibody (blue) and anti-B220 antibody (magenta) on day 7 post-immunization. The white dotted curve was used to distinguish B-cell zone and T-cell zone. Enlarged image was derived from the B-cell zone of left panel. The data are presented as the mean ± s.e.m. Statistical analysis was performed by one-way ANOVA with Fisher’s LSD post-test

Delivering vaccines directly to dLNs has been currently deemed as an opportunity to improve immunotherapeutic effects [7]. However, owing to the anatomy of dLNs, particulate antigens are difficult to effectively enter the lymphatics and travel to dLNs [7]. Considering that abundant branches of lymphatic vessels are located at acupoints [22], we investigated whether ADN could promote the direct delivery of NVs to dLNs. Three-dimensional (3D) in vivo imaging system (IVIS) observation of mice on day 3 after immunization with Cy5.5-labeled NVs was carried out. It was found that ST36 rather than IM administration exhibited a strong fluorescence signal in dLNs (Fig. 3a). The dLN tissues were excised from mice for fluorescence imaging at the predetermined time points after injection. As shown in Figs. 3b, c and S5, IM administration emerged relatively weak fluorescence signals, which disappeared completely with time extending to 48 h. In contrast, ST36 injection enabled rapid yet long-lasting accumulation of NVs in dLNs, which might be ascribed to the densely distributed lymphatic vessels at the acupoint site [22]. Through comprehensive monitoring of the distribution, metabolism, and excretion of Cy5.5-labeled NVs in vivo, we found that NVs injected through both IM and ST36 routes diffused slowly within 3 days post-administration (Fig. S6a, c). In addition, IVIS images of major organs presented a major accumulation of NVs in the liver and kidneys (Fig. S6b, c). It has been reported that peripheric DCs are essential in the entry of NVs into dLNs, as peripheric DCs internalize antigen and subsequently migrate to dLNs directed by the upregulated CCR7 [5]. To ascertain the direct delivery of NVs to dLNs, mice were injected with anti-CCR7 monoclonal antibody (αCCR7) to abolish the migration of immune cells from the periphery to dLNs. Notably, enhanced fluorescence signals in dLNs were comparable in the αCCR7-treated and isotype monoclonal antibody (iso)-treated mice after immunization through ST36 route (Fig. 3d, e), which was further verified by mean fluorescence intensity (MFI) of dLN cell suspension using flow cytometry (Fig. 3f). These data suggested that NVs immunized through ST36 route could directly accumulate into dLNs in a CCR7-independent manner.

To visualize the distribution of NVs in dLNs, the tissues were sliced for CLSM imaging. In comparison with primary retention at the periphery after IM injection, ST36 route enabled far deeper distribution inside dLNs (Fig. 3g, h), reflecting the acupoint-preferred delivery of NVs to dLN paracortex and cortex, where the majority of immune cells reside. Given the fact that acupoints have been identified as a type of neurogenic spot featured by plasma extravasation and vasodilation in postcapillary venules [45], lymphatic vessels at acupoints might also exhibit dilation, contributing to deeper penetration of NVs in dLNs. The strong fluorescence signal in the images of dLN tissue sections from the IM group could be attributed to the accumulation of NVs at the periphery of dLNs, whereas NVs were more dispersed within the interior regions of dLNs in the ST36 group. As expected, flow cytometric analysis displayed a significantly higher proportion of NV-positive cells in the NV-ST36 group than the NV-IM group (Fig. 3i). Ascertainment of NV-positive cells elucidated that NVs preferentially distributed in B-cell zones after immunization through ST36 route compared to IM route (Fig. 3j), which was visualized by using co-staining experiments with antibodies against CD3 and B220 (Fig. 3k). We hypothesized that the deep penetration of NVs into dLNs resulted in the increased distribution of NVs in B-cell zones. In addition, a less proportion of NV-positive barrier cells including medullary cord macrophages (MCM), medullary sinus macrophages (MSM), and subcapsular sinus macrophages (SSM) in the NV-ST36 group confirmed the deeper distribution of NVs inside dLNs (Fig. 3j). Collectively, the diffusion of NVs injected at the acupoint site enabled direct accumulation of NVs to the deep area of dLNs in a CCR7-independent manner, which implied a potential to enhance adaptive immunity.

### Facilitation of Adaptive T-Cell-Mediated Immune Responses in dLNs

**Fig. 4:**
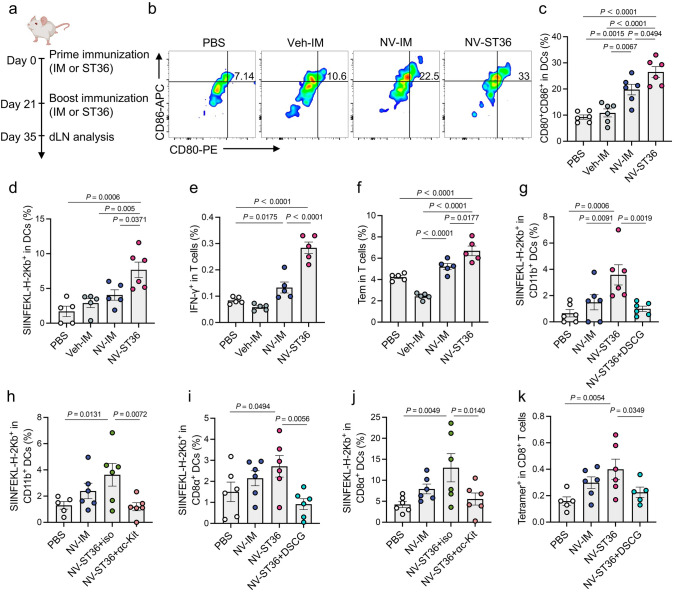
ADN-mediated T-cell immune responses in dLNs.** a** Scheme of immunization and sample collection. Mice were immunized with NVs on days 0 and 21. Then, mice were euthanatized, and LN tissues were collected to evaluate immune responses 35 days post-immunization (*n* = 5 or 6). **b** Representative flow cytometry scatter plots and **c** statistical data of CD80^+^CD86^+^ mature DCs in dLNs.** d** Percentage of SIINFEKL^+^ antigen-presenting DCs in dLNs. **e** Percentage of IFN-γ^+^ in T cells in dLNs. **f** Percentage of Tem cells (CD62L^−^CD44^hi^) in total T cells. **g**-**k** Mice were injected with PBS or NVs through IM or ST36 route (*n* = 6). **g**, **i**, **k** Mice immunized through ST36 additionally received intraperitoneal injection with PBS or DSCG. **h**,**j** Mice immunized through ST36 additionally received intraperitoneal injection with c-kit antibody or isotype control. The dLNs were collected at 24 h post-immunization to detect immune responses. Percentages of SIINFEKL^+^ antigen-presenting **g**, **h** CD11b^+^ DCs and **i**, **j** CD8α^+^ DCs in dLNs. **k** Percentage of tetramer^+^ CD8^+^ T cells in dLNs. The data are presented as the mean ± s.e.m. Statistical analysis was performed by one-way ANOVA with Fisher’s LSD post-test

As amplified innate responses at the injection site and enhanced access to dLNs are both pivotal for NVs to initiate adaptive immune responses, we next studied whether ADN could improve immunization effectiveness. The T-cell-mediated immune responses in dLNs of mice immunized with NVs by IM or ST36 route on days 0 and 21, respectively, were analyzed (Fig. 4a). PBS and PEI vehicle (termed Veh-IM) injected to IM region were set as controls. Given enhanced recruitment of CD11b^+^ DCs at the injection site and their migration to dLNs to transfer antigens to dLN-resident DCs or present to T cells, we first detected the distribution and activation of DCs in dLNs. After administration by IM or ST36 route, the total number of CD11c^+^ DCs was assessed on days 1, 2, 3, and 7. In comparison with IM route, mice exhibited the most dramatic increase of total number of CD11c^+^ DCs in dLNs on day 3 post-immunization (Fig. S7). Encouragingly, the percentage of mature DCs in mice treated with NVs increased significantly in the ST36 group compared to that of the IM group (Fig. 4b, c). The antigen presentation ability of DCs was detected to evaluate T-cell-mediated immune responses. As depicted in Fig. 4d, ST36 injection resulted in the highest level of SIINFEKL-H-2 Kb^+^ DCs, elucidating the enhanced antigen presentation. It has been reported that CD4^+^ T-cell subset provides necessary help for longer-lived antibody responses and high-avidity tumor antigen-specific CD8^+^ T-cell expansion [46, 47]. Consistently, ST36 injection induced a CD4-biased response, which might be ascribed to the incorporation of the vehicle (Fig. S8a). Furthermore, in the ST36 group, CD4^+^ T cells shifted their functions toward T helper (Th) 1 responses by increasing interferon-gamma (IFN-γ) production (Figs. 4e and S9a) and elevating the ratio of IFN-γ/interleukin-4 (IL-4)-producing CD4^+^ T cells (Fig. S8b). Treatment with NVs by ST36 injection also induced the highest levels of IFN-β and IFN-γ in serum among all groups (Fig. S10), suggesting a potential in eliciting antiviral and antitumor effects. Note that memory T cells contain distinct populations of central memory (Tcm) and effector memory (Tem) cells, in which Tcm cells mainly home to dLNs and differentiate to effector T cells in response to antigenic stimulation, whereas Tem cells rapidly migrate to inflamed tissues and display an immediate effector function [48]. As plotted in Figs. 4f and S9b, the proportion of Tem cells in the ST36 group was notably greater than those in other groups.

Interestingly, only ADN resulted in an obvious increment in the proportion of SIINFEKL^+^ antigen-presenting CD11b^+^ DCs in dLNs (Figs. 4g, h and S11), which could be attributed to the migration of antigen-presenting CD11b^+^ DCs from the injection site to dLNs. CD8α^+^ DCs, which are dLN-resident DCs and more efficient for presentation [49], also presented the highest level of SIINFEKL peptide on the surface in the NV-ST36 group (Figs. 4i, j and S11). Moreover, either ablation of mast cells or inhibition of mast cell activation blunted the elevated proportions of antigen-presenting CD11b^+^ DCs and CD8α^+^ DCs triggered by ADN (Fig. 4g-j). More importantly, ADN stimulated the highest percentage of OVA-specific (tetramer^+^) T cells in dLNs, which was markedly reversed by inhibiting mast cell activation (Figs. 4k and S9c). We also found a significantly higher percentage of IFN-γ-producing CD8^+^ CTLs in the ST36 group, with a slight decrease after inhibition of mast cell activation (Fig. S12), suggesting an enhanced T-cell effector function and cytotoxicity. These results reasoned that the mast cell-activated niche at acupoints could promote antigen presentation by dLN-resident DCs, thereby amplifying antigen-specific T-cell immune responses.

### Amplification of Adaptive B-Cell-Mediated Immune Responses in dLNs

**Fig. 5:**
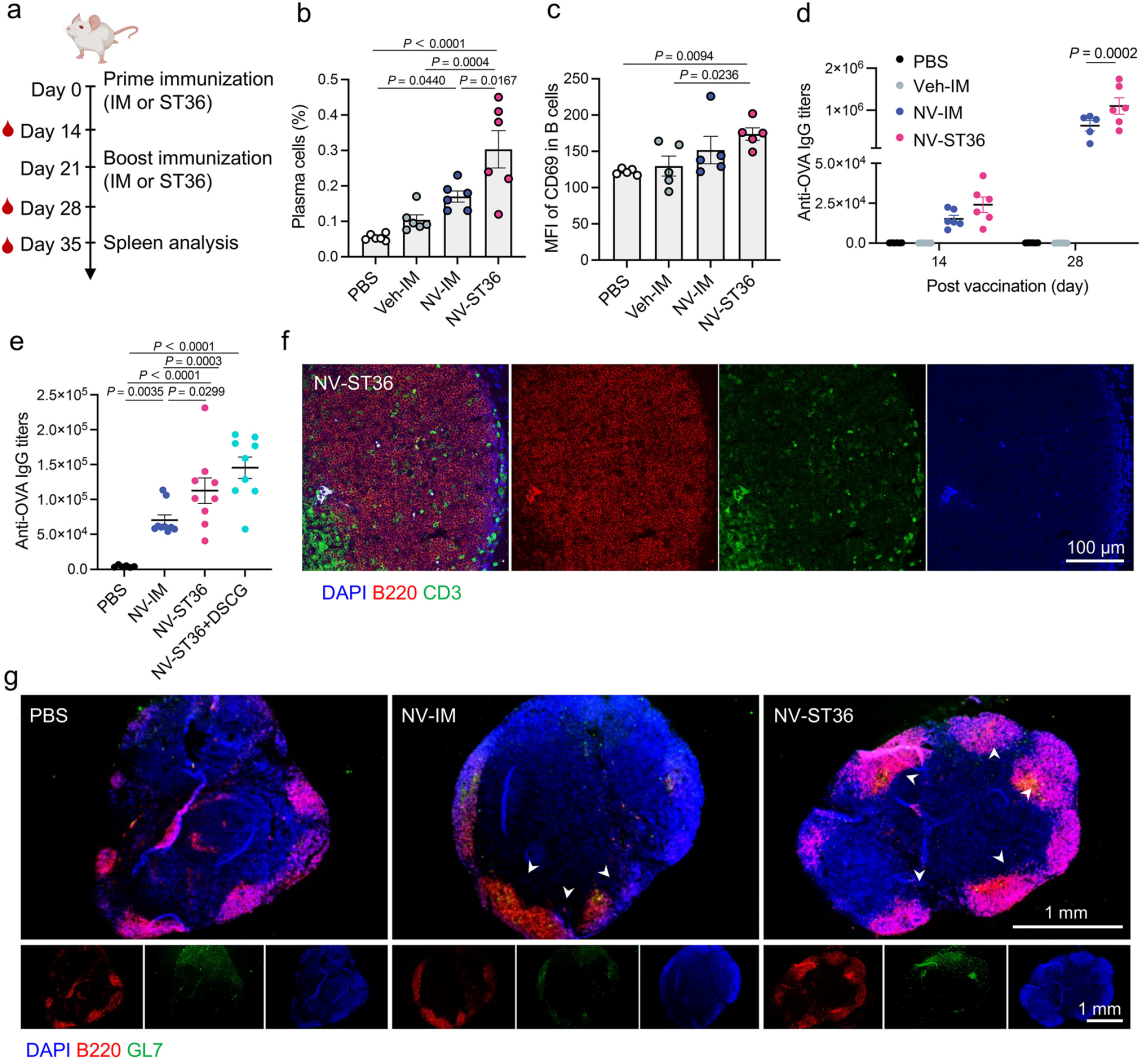
ADN-mediated B-cell immune responses in dLNs.** a** Scheme of immunization and sample collection. Mice were immunized with NVs on days 0 and 21. Then, mice were euthanatized, and dLN tissues were collected to evaluate immune responses 35 days post-immunization. Red drop symbols represent the time points at which serum samples were collected for antibody detection (*n* = 6). **b** Percentage of plasma cells (CD138^+^B220^−^) in dLNs. **c** MFI quantification of CD69 on B cells in dLNs. **d** Kinetics of OVA-specific IgG antibody titers in serum assessed by ELISA. **e** Level of OVA-specific IgG antibody titers in serum on day 35 post-immunization. **f** Representative images of dLN sections stained with anti-B220 antibody (red), anti-CD3 antibody (green), and DAPI (blue) on day 2 post-immunization. **g** Representative immunofluorescence images of dLN sections stained with anti-B220 antibody (red), anti-GL7 antibody (green), and DAPI (blue) on day 5 post-immunization. White arrows represent GCs. The data are presented as the mean ± s.e.m. Statistical analysis in **d** was performed by two-way ANOVA with the Bonferroni correction post-test. Statistical analysis in **b**, **c**, **e** was performed by one-way ANOVA with Fisher’s LSD post-test

Next, we attempted to evaluate the influence of ADN on B-cell-mediated immune responses in dLNs collected from mice immunized with NVs by IM or ST36 route on days 0 and 21, respectively (Fig. 5a). Pathogen-specific antibodies are produced by plasma cells, which are the terminal differentiation form of B cells encountering antigens [50]. ST36 injection elicited a strong differentiation of B cells into antibody-secreting plasma cells in dLNs (Fig. 5b). In line with this, expression of activation marker CD69 on B cells was remarkably upregulated in the ST36 group than those of other controls (Fig. 5c). After prime immunization, enzyme-linked immunosorbent assay (ELISA) demonstrated that the serum from immunized mice appeared a detectable level of OVA-specific IgG, and a slight increase was observed in the ST36 group compared to the IM group (Fig. 5d). This difference in OVA-specific IgG titers between the IM and ST36 groups was further significantly amplified after a boosting dose (Fig. 5d). The elevated level of circulating antibodies indicted that ADN induced a robust protective immune response. Of note, inhibiting mast cell activation did not lead to a reduction in the elevated level of OVA-specific IgG induced by ADN (Fig. 5e). We, therefore, hypothesized that the direct delivery of NVs into dLNs rather than mast cell activation at acupoints was mainly involved in the expression of OVA-specific IgG. GC reactions in dLNs are essential to produce high affinity antibodies and a long-lived T-cell-dependent humoral immunity [51]. In addition, it is known that the interaction of follicular helper T (Tfh) cells with B cells is required for GC formation [51]. In line with this notion, a large amounts of T cells were observed in B-cell zones after immunization via ST36 route (Figs. 5f and S13), implying the potential of enhanced GC reactions. As such, dLNs from mice treated with NVs by ST36 route were collected and stained. As presented in Fig. 5g, immunofluorescence images illustrated a dramatic increase in GC formation compared to that of IM route, which indicated an enhanced GC differentiation of B cells. These results suggested that ADN promoted adaptive B-cell-mediated immune responses in dLNs. Additionally, we interrogated whether ADN could influence the activation of other immune cells, such as natural killer (NK) cells and macrophages. As shown in Fig. S14, no significant differences were observed between the IM and ST36 groups regarding the percentage of IFN-γ^+^ NK cells and the polarization of type 1 macrophages (M1) cells.

### Induction of Robust Systemic Immune Responses

**Fig. 6:**
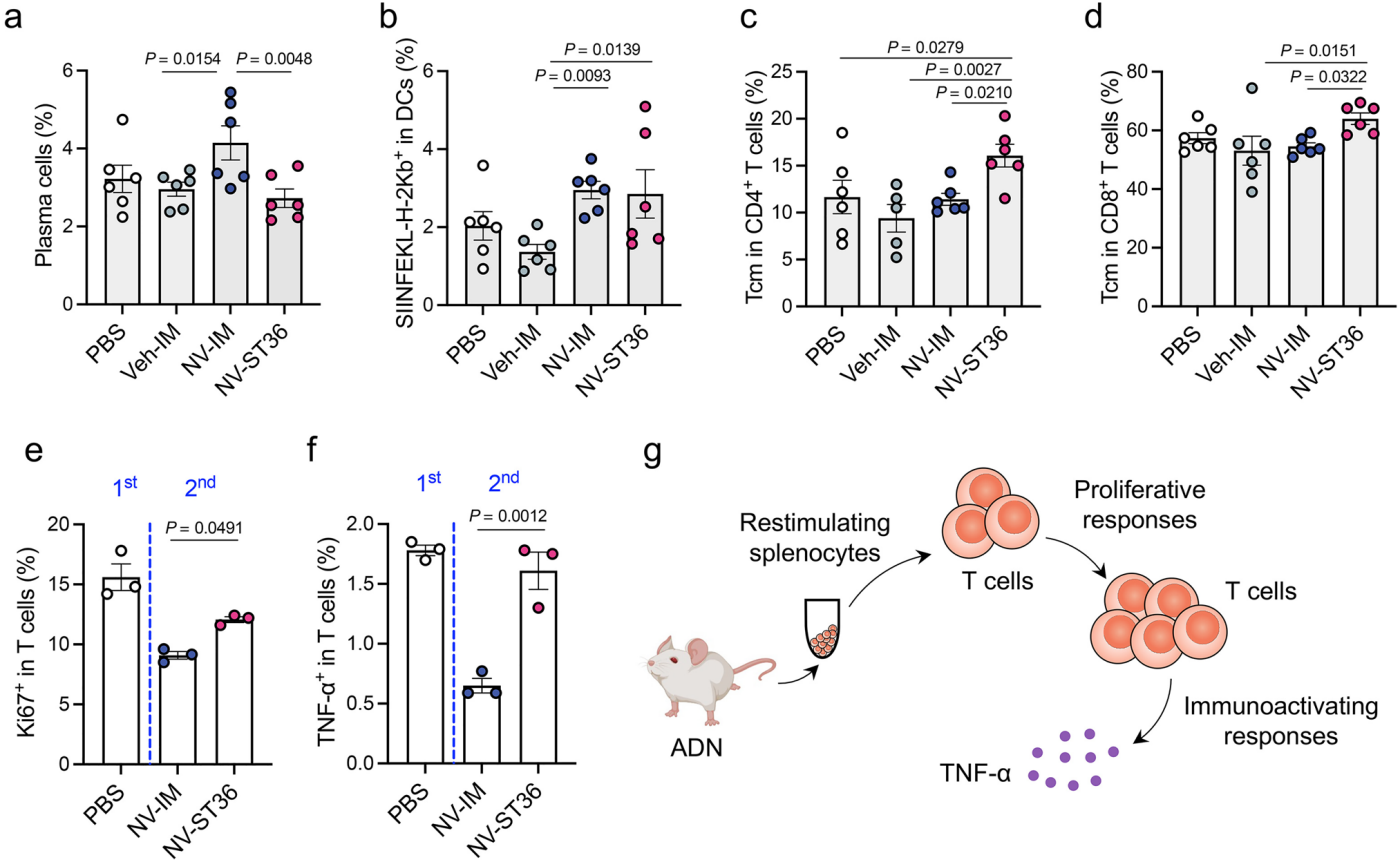
ADN-induced systemic immune responses in mice.** a**-**d** Mice were immunized with NVs on days 0 and 21. Then, mice were euthanatized, and spleen tissues were collected to evaluate immune responses 35 days post-immunization (*n* = 6). Percentages of **a** plasma cells (CD138^+^B220^−^) and **b** SIINFEKL^+^ antigen-presenting DCs in the spleen. Percentages of Tcm cells (CD62L^+^CD44^hi^) in **c** CD4^+^ T cells and **d** CD8^+^ T cells, respectively. **e**, **f** Splenocytes from mice immunized with (2nd) or without (1st) NVs were isolated and co-cultured with NVs for 48 h (*n* = 3). Percentages of **e** Ki67^+^ and **f** TNF-α^+^ in T cells. **g** Schematic diagram of ADN-enabled prevention of T-cell anergy. ADN promotes T-cell proliferation and TNF-α secretion upon restimulation with previous antigen. The data are presented as the mean ± s.e.m. Statistical analysis was performed by one-way ANOVA with Fisher’s LSD post-test

We then turned our attention to explore the benefits of ADN by asking whether it could elicit a systemic and long-term immunity. The spleen, as a gatekeeper of systemic immunity, plays a primary role in initiating immune responses against blood-borne antigens [52]. Induction of systemic immunity via ADN was thus verified by detecting immune responses in the spleen of immunized mice. Unexpectedly, ADN exhibited less plasma cells in the spleen compared to the IM group (Fig. 6a), indicating that anti-OVA antibody in serum was mainly derived from dLNs. Although antigen presentation by DCs was comparable (Fig. 6b), the percentages of Tcm cells in CD4^+^ and CD8^+^ T cells collected from mice treated by ST36 injection showed a significant increase than those in all other groups (Fig. 6c, d), suggesting rapid proliferation and differentiation of antigen‐specific effector T cells. Thus, we speculated that ADN could markedly inhibit immune tolerance by counteracting T-cell anergy, which is hyporesponsive to subsequent antigen encounter. To assess the prevention of T-cell anergy by ADN, splenocytes from mice immunized without (1st) or with (2nd) NVs were isolated and stimulated with NVs in vitro. Compared to the 1st group, the expression of Ki67, a marker for cell proliferation, on T cells was much lower in the 2nd group, indicating a hypoproliferation of antigen-experienced T cells following secondary stimulation (Fig. 6e). However, antigen-experienced T cells in the ST36 group expressed a much higher level of Ki67 in response to a secondary stimulation compared to that in the IM group. Similarly, in comparison with IM injection, ST36 route achieved an obvious increment in tumor necrosis factor-alpha (TNF-α)-secreting T cells after restimulation (Fig. 6f). We hypothesized that upon re-exposure to the antigen, the ADN-induced protective immune cells generated in dLNs could rapidly migrate to infected tissues, while the protective response elicited by ADN in the spleen could preferentially drive effector T-cell differentiation. Taken together, ADN triggered robust systemic immune responses, in which antigen-experienced T cells in the spleen retained proliferative and immunoactive to secondary antigen stimulation (Fig. 6g).

### Versatility of ADN

Having confirmed the improved adaptive immunity both in dLNs and spleen, we further validated the versatility of ADN by replacing different nanocarriers, cargos, and acupoint. Our previous study showed that in situ polymerization of dopamine could be applied to fabricate NVs that simultaneously carry antigen and agonist by Michael addition and Schiff base reaction-mediated conjugation between catechol groups in polydopamine nanoparticles (PDA-NPs) and nucleophilic primary amines and thiols in cargo molecules [34]. For proof-of-principle, we first chose S1 protein and R848 as antigen and immunoadjuvant, respectively. S1 subunit of SARS-CoV-2 spike protein has been identified as a sophisticated antigen that induces neutralizing antibodies against virus infection [53], while R848, an agonist of toll-like receptor 7 and 8 (TLR7/8), emerges as a potent driver of priming innate immunity against infectious disease, cancer, and autoimmune disorders [37, 54, 55]. To this end, NVs co-loaded with S1 protein and agonist (termed as S1/R848 NVs) were fabricated (Fig. S15a). DLS measurement showed that average hydrodynamic size of S1/R848 NVs was approximately 40–50 nm larger than those of PDA-NPs (Fig. S15b), while the surface zeta potential was about 18 mV lower (Fig. S15c). The loading of antigen was evidenced by flow cytometric analysis, showing a significant increment in fluorescence intensity of NVs loaded with S1-Cy5.5 and R848 (Fig. S15d). The change in S1 protein concentration in the reaction mixture following polymerization was used to determine the loading content of antigen, which was calculated to be 54 wt% by optimizing its feeding concentration (Fig. S15e).

**Fig. 7:**
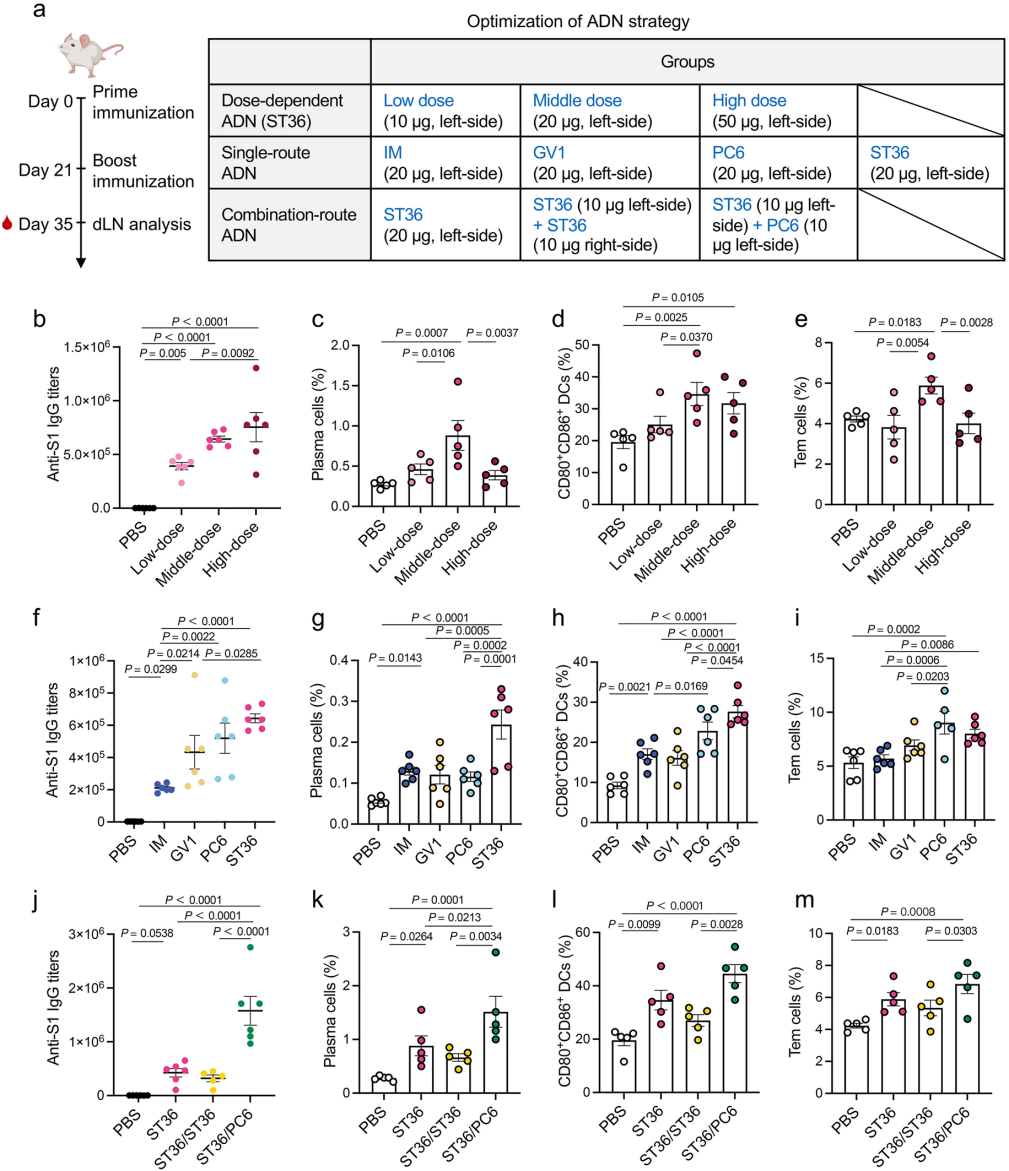
Versatility of ADN.** a** Scheme of experimental design for detecting S1-specific adaptive immunity and optimizing immunization strategy (*n* = 6). Dose-dependent ADN: immunization with S1/R848 NVs (10, 20, or 50 μg S1 protein) by ST36 route; single-route ADN: immunization with S1/R848 NVs by IM, GV1, PC6, or ST36 route; combination-route ADN: ST36 injections in both sides or left-side ST36 plus left-side PC6. **b**, **f**, **j** Levels of IgG antibody in serum assessed on day 35 following **b** dose-dependent ADN, **f** single-route ADN, and (**j**) combination-route ADN, respectively. **c**, **g**, **k** Percentages of plasma cells (CD138^+^B220^−^) in dLNs from mice following **c** dose-dependent ADN, **g** single-route ADN, and **k** combination-route ADN, respectively. **d**, **h**, **l** Percentages of CD80^+^CD86^+^ mature DCs in dLNs from mice following **d** dose-dependent ADN, **h** single-route ADN, and (**l**) combination-route ADN, respectively.** e**, **i**, **m** Percentages of Tem cells (CD62L^−^CD44^hi^) in dLNs from mice following **e** dose-dependent ADN, **i** single-route ADN, and **m** combination-route ADN, respectively. The data are presented as the mean ± s.e.m. Statistical analysis was performed by one-way ANOVA with Fisher’s LSD post-test

After prime-boost immunization on days 0 and 21, serum samples were collected on day 35 to evaluate S1-specific IgG antibody titers by ELISA (Fig. 7a). In view of that the effectiveness of vaccines is often dose-dependent, we immunized mice with different doses of S1/R848 NVs, including S1 doses of 10 μg (low dose), 20 μg (middle dose), and 50 μg (high dose) via ST36 injection, respectively. Note that even low-dose immunization by ST36 injection could significantly elicited S1-specific IgG titers compared to middle-dose immunization by IM route (Fig. 7b, f). There were higher S1-specific IgG titers in high-dose group than in low-dose group, and no significant difference was found between middle- and high-dose groups (Fig. 7b). Interestingly, middle-dose group triggered the highest levels of proliferation of plasma cells, maturation of DCs, and differentiation of Tcm cells (Fig. 7c-e). We reasoned that the lower percentage of plasma cells and Tcm cells in high-dose group compared to in middle-dose group might be attributed to the fact that high-dose antigens favor clonal deletion [56]. These results suggested that administration with middle-dose S1/R848 NVs was an optimal condition for ADN. Next, we further explored whether ADN could promote adaptive immunity through other acupoint route. Intriguingly, ADN through PC6 and ST36 routes could both promote the accumulation of NVs in dLNs, reflected by the substantially enhanced fluorescence intensity in dLNs even with time prolonging up to 48 h (Fig. S16a). Corresponding radar charts further showed the preference of NVs to accumulate in dLNs after acupoint injection via PC6 and ST36 (Fig. S16b). This observation suggested that the enhanced delivery of NVs into dLNs might be independent of the chemical and physical features of NVs. As exhibited in Fig. 7f, GV1, PC6, and ST36 injections with S1/R848 NVs both induced higher antibody titers than IM route. All immunized mice presented significantly increased percentage of plasma cells, with the highest level in the ST36 group (Fig. 7g). Of note, immunization by PC6 and ST36 injections was able to increase DC maturation and also generated the greatest amount of Tem cells compared to other routes (Fig. 7h, i). Furthermore, we evaluated the efficacy of combination-route ADN, in which mice were immunized with equivalent S1/R848 NVs by unilateral ST36, bilateral ST36, or unilateral PC6 plus ST36 route. Markedly, S1/R848 NVs induced significant high levels of S1-specific IgG (Fig. 7j), percentages of plasma cells, mature DCs, and Tem cells (Fig. 7k-m) in the combination-route group in contrast with single-route immunized mice. Consistently, the combination-route group showed the strongest Th1-bias response by producing a higher level of IFN-γ than other groups (Fig. S17), indicating the ability to effectively eradicate intracellular pathogens. We hypothesized that the facilitated efficacy of combination-route ADN might be ascribed to the synergistic activation of dLNs located at distinct anatomical sites in mice. These data expounded that ADN could improve immunization efficacy through dose- and acupoint route-dependent manners. In addition, safety assessments using hematoxylin and eosin (H&E) staining certified undetectable damages to major organs in immunized mice (Fig. S18), supporting favorable safety of ADN. Collectively, these results proposed that ADN displaying clinically translational potential could be applied as a universal strategy for diverse nanovehicles and antigenic cargos.

### Elicitation of Effective Antitumor Immune Responses

**Fig. 8:**
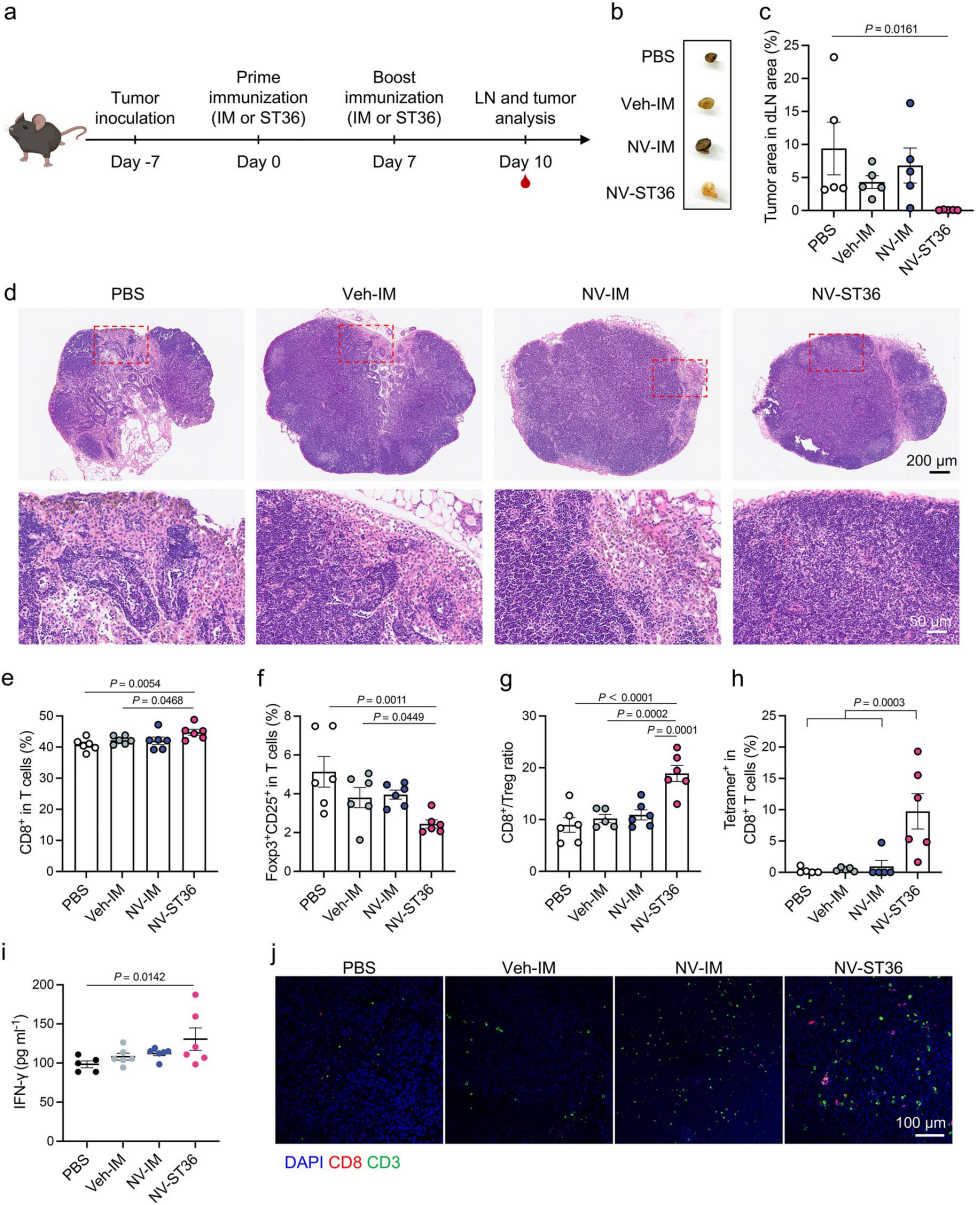
ADN-induced antitumor responses. **a** Scheme of experimental design of assessing ADN-induced antitumor responses in a mouse model of B16-OVA melanoma (*n* = 6). The dLNs and tumor tissues were collected on day 10. Red drop symbols represent serum sampling time points. **b** Digital photos of the sampled dLNs. **c** Percentage of LN area occupied by tumor lesions based on **d** LN histopathological analysis. Percentages of **e** CD8^+^ T cells and **f** Treg cells (Foxp3^+^CD25^+^CD4^+^) in dLNs. **g** Ratio of CD8^+^ T cells to Treg cells in dLNs. **h** Percentages of tetramer^+^ CD8^+^ T cells in dLNs. **i** Level of IFN-γ in serum measured by ELISA. **j** Representative immunofluorescence images of tumor sections staining with anti-CD8 antibody (red) and anti-CD3 antibody (green). Cell nuclei were stained with DAPI (blue). The data are presented as the mean ± s.e.m. Statistical analysis was performed by one-way ANOVA with Fisher’s LSD post-test

Having confirmed the versatility of ADN, we further assessed the values of ADN in tumor-bearing mice. The efficacy of ADN was evaluated in a murine model of B16-OVA melanoma (Fig. 8a). Tumor-bearing mice were immunized with PBS, PEI vehicle, and NVs through IM and ST36 route, respectively. Tumor size and body weight fluctuation of each mouse were closely monitored after immunization. Clearly, tumor growth was largely suppressed in mice immunized with NVs via ST36 injection compared to IM injection (Fig. S19a). In addition, negligible body weight fluctuation was observed for all treat mice during the monitoring period (Fig. S19b). Considering that spreading to dLNs is an important marker of poor prognosis in cancer patients [57], dLNs were collected for analyzing metastasis. As shown in Fig. 8b, the presence of melanoma cells was clearly observed in tumor-bearing mice, while treatment with NVs by ST36 route resulted in the lowest dLN metastases among all treated groups. H&E staining further confirmed the reduction of metastatic lesions in dLNs (Fig. 8c, d). Mechanistically, LN colonization by tumor cells is deemed pivotal for distant metastasis due to the generation of tumor-specific immune tolerance, featured by induction of regulatory T (Treg) cells and reduction of immune cytotoxicity [57]. Compared to the PBS control, treatment with NVs through ST36 route significantly increased the percentages of total T cells (Fig. S19c) and CTLs (Fig. 8e), while decreased the frequency of Treg cells (Fig. 8f), which were not found in all other treated groups. Furthermore, ADN led to a higher ratio of effector CTLs to Treg cells (Fig. 8g), supporting that ADN could effectively reduce LN metastasis-mediated immune tolerance. To certify antitumor immune responses enabled by ADN, tumor antigen-specific CTLs in dLNs were evaluated. In contrast with all control groups that had a similar level, the frequency of tetramer^+^ CTLs in dLNs displayed a sixfold increment in the ST36 group (Fig. 8h), verifying a potent tumor-specific T-cell immune response. ADN also achieved the highest level of IFN-γ in serum, which referred to a systemic antitumor immune response (Fig. 8i). Strikingly, immunofluorescence staining clarified that the infiltration of CTLs in the tumor tissue of mice treated with NVs via ST36 injection dramatically increased, further implying the generation of a potent antitumor immunity (Fig. 8j). As claimed in Fig. S19d, H&E staining displayed the largest necrotic area in tumor tissue from the ST36 group, affirming efficient tumor regression. Lastly, to assess the protective effectiveness induced by ADN, mice immunized with or without NVs by ST36 injection were challenged with B16-OVA cells in a prophylactic study (Fig. S20a). Surprisingly, ADN resulted in complete prevention against tumor occurrence in all inoculated mice (Fig. S20b, c). Briefly, the above data elucidated that ADN remarkedly suppressed tumor growth via reducing LN metastasis and enhancing antitumor immune responses. Given the importance of dLNs in the ADN-mediated tumor suppression, we speculated that the delivery of NVs through the tumor-proximal acupoint might contribute to the therapeutic benefit.

## Conclusions

Immunization with NVs by conventional approaches often results in insufficient activation of innate immune cells at the injection site and poor delivery of antigens to dLNs, which severely limit the rapid and potent induction of adaptive immune responses. Previous improvements mainly depend on the functionalization of biological and synthetic nanomaterials, which are designed with a given or combined functional motif to elicit strong adaptive immune responses [10]. For instance, a wide range of NVs have been developed to co-deliver immunoadjuvants, release antigen and/or immunoactivators sustainedly, and enhance targeting ability, with aims to promote the recruitment of tissue-resident immune cells, the capture and internalization of antigens by APCs, and the accumulation of vaccines in dLNs [7, 10]. However, previous approaches solely generate single-niche immunological priming either at the injection site or in dLNs and their effectiveness remains unsatisfactory. In this work, we report a new strategy of dual-niche immunological priming, which is enabled by ADN. Acupoint injection not only forms an in situ immunological niche at the injection site, but also increases the accumulation of NVs in dLNs and forms the second immunological niche.

We demonstrate that a large number of mast cells reside in acupoint region and ADN can activate these mast cells to release mediators and upregulate CD40L expression. Subsequently, the activation of mast cells enhances tissue-resident CD11b^+^ DC recruitment and their antigen presentation, accompanied by the elevated expression of CD40 on DCs. As CD40 signaling is required for DC activation, we speculate that mast cells could preferentially communicate with CD11b^+^ DCs through CD40-CD40L interaction. These CD11b^+^ DCs furtherly migrate to dLNs to facilitate the transfer of antigens to dLN-resident CD8α^+^ DCs and T-cell priming. On the other hand, ADN enables the enhanced delivery of NVs into dLNs, which is not blunted after abolishing the migration of immune cells to dLNs by CCR7. We, therefore, reason that NVs can directly drain to dLNs due largely to the enriched location of lymphatic system at acupoint regions. It is worth mentioning that the inhibition of mast cell activation blunts the elevated proportions of antigen-presenting CD11b^+^ DCs and CD8α^+^ DCs as well as antigen-specific T cells triggered by ADN, while it does not attenuate the increased expression of antigen-specific IgG antibody. This result suggests that in situ-forming immunological niche at the injection site is primarily involved in the improved T-cell immune responses in dLNs, yet the formed immunological niche in dLNs mainly contributes to the enhanced B-cell immune responses in dLNs. Strikingly, ADN prevents splenic T-cell anergy by priming immunization and maintaining T cells in an “on” status against the secondary antigen stimulation, which is conducive to eliminating the pathogens from circulation.

We find that the effectiveness of ADN can be further enhanced by using different acupoints, varying NV dose, and combining dual acupoints. This strategy is also applicable for different delivery nanocarriers and antigenic cargos. As proof-of-concept applications, ADN is capable to boost the secretion of virus-specific IgG titers by inducing the high levels of proliferation of plasma cells, maturation of DCs, and differentiation of Tcm cells. Meanwhile, ADN effectively inhibits the growth of established melanomas and prevents melanomas occurrence and metastasis by eliciting antigen-specific T-cell responses and increasing the ratio of CTLs to Treg cells. Impressively, the enhanced effectiveness of ADN is observed in both C57BL/6 and BALB/c strains with different immune backgrounds. Although we have attempted to optimize injection dose and sites and the combination of dual acupoints, more schemes need to be carefully evaluated to confirm efficacy and safety, particularly in multiple preclinical models before considering for further translation. Moreover, additional control groups, such as the delivery of NVs via intranodal, subcutaneous, or intravenous route need to be involved to verify the efficacy of ADN in the future studies. Although our findings suggest that both PEI-based NVs and PDA-based NVs injected through the ST36 route can elicit higher T-cell and B-cell responses than those of the IM route, a broader range of antigens and nanoparticle carriers need to be assessed to validate the generalizability of the ADN strategy. Manipulation skills, including intensity, depth, and repetition, are also pivotal to achieve appropriate acupoint stimulation, which potentially influences the efficacy of ADN. In addition, the selection of suitable immune adjuvants incorporated into NVs is instrumental in elevating the effectiveness of ADN. In summary, this work, for the first time, unveils the significance of acupoint stimulation in nanovaccination and opens a window for developing simple yet versatile approaches to enhance the antiviral and antitumor efficacies of various NVs.

## Supplementary Information

Below is the link to the electronic supplementary material.

Supplementary file1 (DOCX 45512 KB)
