# Supplementary material for: Eliciting Dual-Niche Immunological Priming by Acupoint Delivery of Nanovaccines
Source: Nanomicro Lett. 2025 May 28;17:280. doi: 10.1007/s40820-025-01789-y (PMC12119423; doi:10.1007/s40820-025-01789-y)
Supplement: Supplementary file 1 — Supplementary file1 (DOCX 45512 KB) [file 40820_2025_1789_MOESM1_ESM.docx]

Supporting Information for

**Eliciting Dual-Niche Immunological Priming by Acupoint Delivery of Nanovaccines**

Lu Wang^1#^, Yanhong Sun^2,3#^, Meiling Yan^3#^, Lihua Wang^2,6#^, Yiyang Wang^3^, Mengmeng Zhang^1^, Qian Li^4^, Huangan Wu^5^, Jinyao Liu^1^*, Chunhai Fan^1,4^*

^1^Shanghai Key Laboratory for Nucleic Acid Chemistry and Nanomedicine, Institute of Molecular Medicine, State Key Laboratory of Oncogenes and Related Genes, Shanghai Cancer Institute, Renji Hospital, School of Medicine, Shanghai Jiao Tong University, Shanghai 200127, P. R. China

^2^Institute of Materiobiology, Department of Chemistry, College of Science, Shanghai University, Shanghai 200444, P. R. China

^3^Division of Physical Biology and Bioimaging Center, Shanghai Synchrotron Radiation Facility, CAS Key Laboratory of Interfacial Physics and Technology, Shanghai Institute of Applied Physics, Chinese Academy of Sciences, Shanghai 201800, P. R. China

^4^School of Chemistry and Chemical Engineering, Frontiers Science Center for Transformative Molecules and National Center for Translational Medicine, Shanghai Jiao Tong University, Shanghai 200240, P. R. China

^5^Key Laboratory of Acupuncture and Immunological Effects, Shanghai University of Traditional Chinese Medicine, Shanghai 200030, P. R. China

^6^Jiaxing Key Laboratory of Biosemiconductors (A), Xiangfu Laboratory, Jiashan 314102, P. R. China

^#^Lu Wang, Yanhong Sun, Meiling Yan, and Lihua Wang contributed equally to this work.

***Corresponding authors. E-mail: [fanchunhai@sjtu.edu.cn](mailto:fanchunhai@sjtu.edu.cn) (Chunhai Fan); [jyliu@sjtu.edu.cn](mailto:jyliu@sjtu.edu.cn) (Jinyao Liu)

**Supplementary Figures**


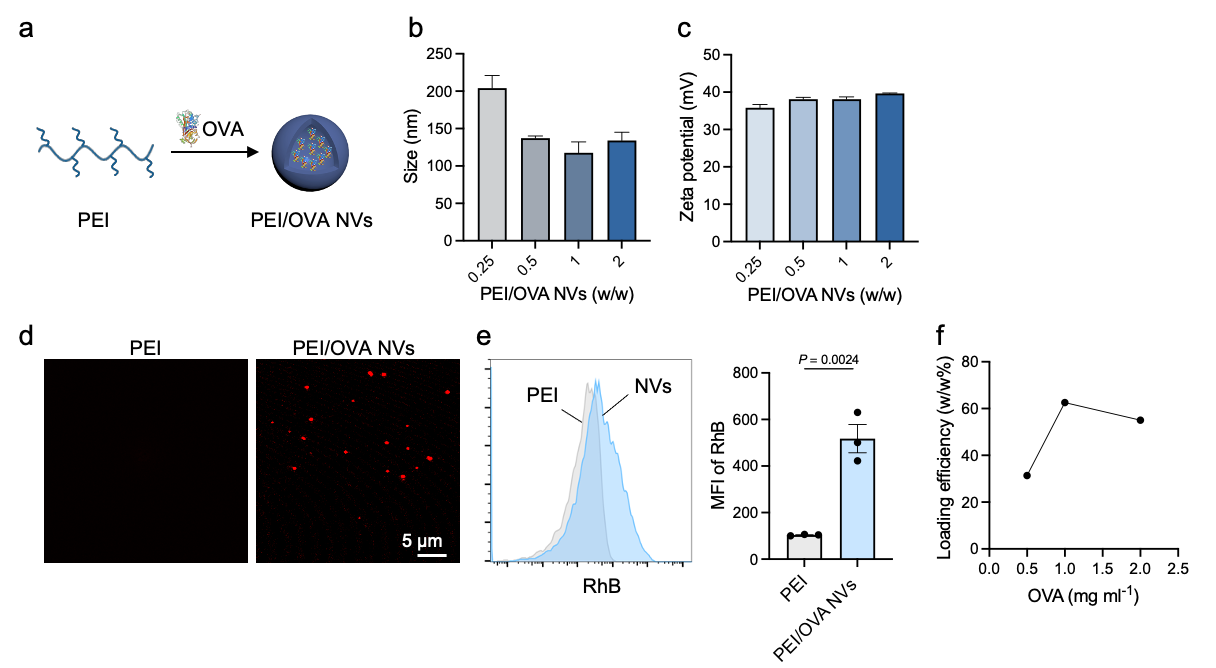


**Fig. 1** **a**, Preparation of PEI/OVA NVs by nanocomplexing. **b**, **c**, DLS measurements of (**b**) size distribution and (**c**) zeta potential of PEI/OVA NVs prepared at different PEI/OVA ratios (w/w). **d**, Representative CLSM images of PEI and PEI/OVA NVs, respectively. **e**, Flow cytometry histograms and statistic results of PEI vehicle and PEI/OVA-RhB NVs. **f**, Loading efficiencies of OVA-FITC in NVs at different feed concentrations measured by a fluorescence microplate assay. The data are presented as the mean ± s.e.m (*n* = 3). Statistical analysis was performed using Student’s *t*-test.
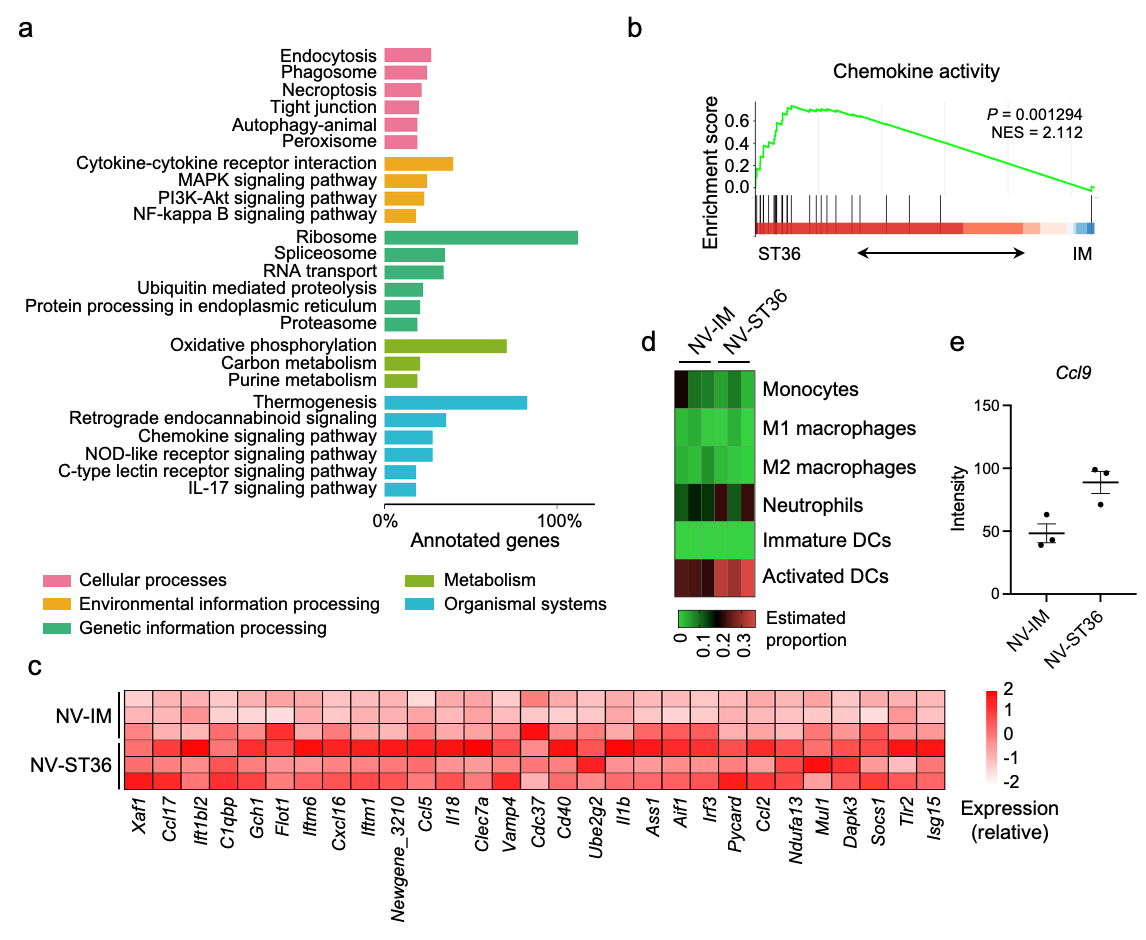


**Fig. 2 a**, KEGG pathway enrichment analysis of genes significantly upregulated in the NV-ST36 group compared to the NV-IM group. **b**, GSEA showing enrichment of genes of chemokine activity pathways. **c**, Heat map showing relative expression of upregulated individual genes related to interferon pathway. **d**, Heat map showing the fraction of several immune cell types infiltrated into the injection site. **e**, Relative expression of *Ccl9* in the NV-IM and NV-ST36 groups determined by transcriptomic analysis (*P* value = 5.91 × 10^-16^)


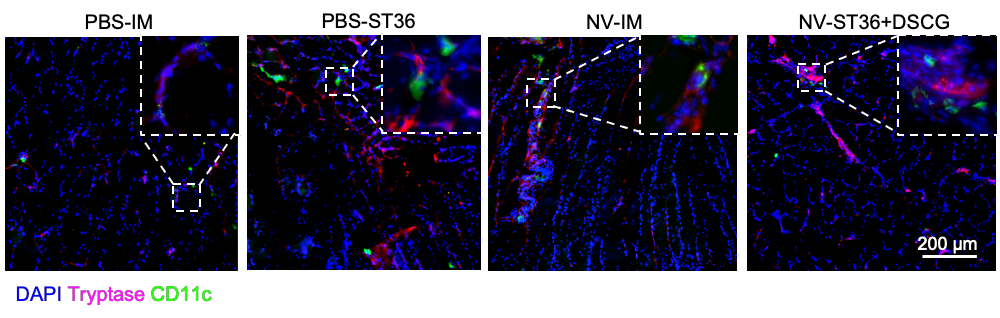


**Fig. 3** Representative immunofluorescence images of tissue sections from IM or ST36 stained with anti-Tryptase antibody (magenta), anti-CD11c antibody (green), and DAPI (blue) at day 3 post-immunization. Enlarged images were derived from the white dotted squares.


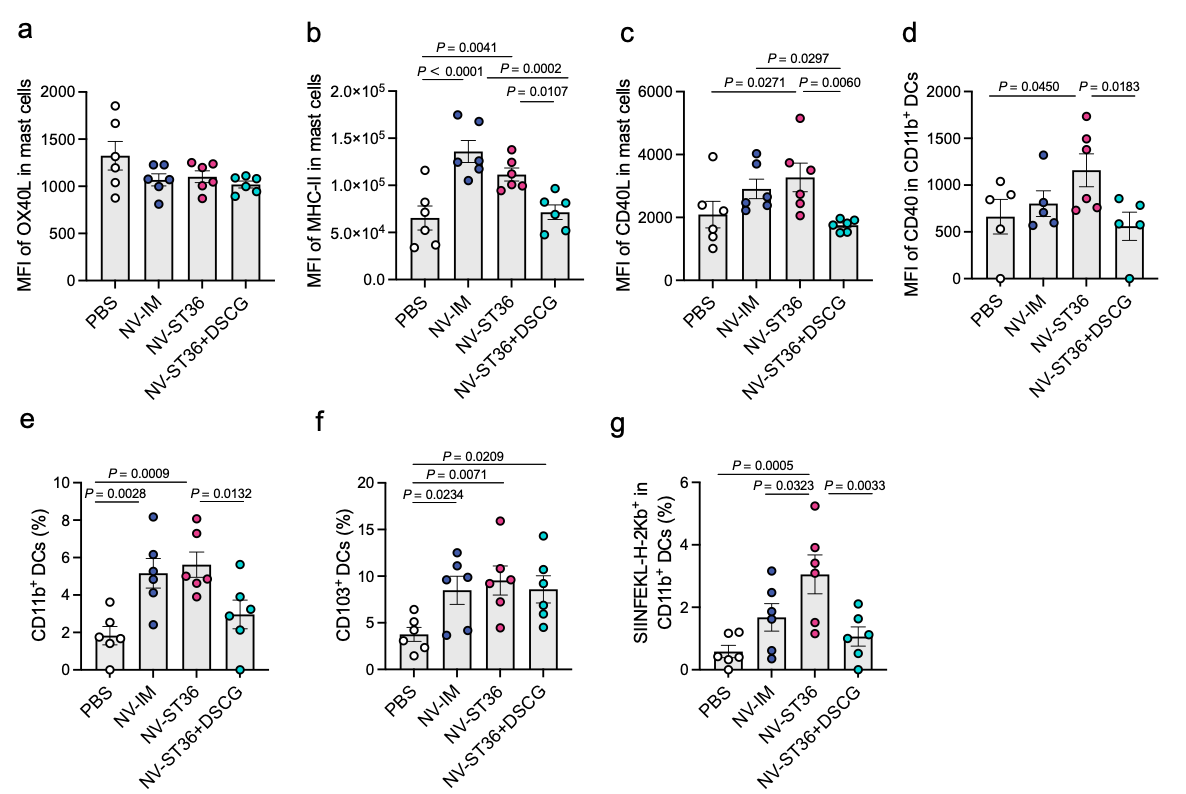


**Fig. 4 a-c**, MFI quantification of (**a**) OX40L, (**b**) MHC-II, and (**c**) CD40L on mast cells in the tissues from the injection site. **d**, MFI quantification of CD40 on the CD11b^+^ DCs in dLNs. **e**-**g**, Percentages of (**e**) CD11b^+^ DCs, (**f**) CD103^+^ DCs and (**g**) SIINFEKL^+^ antigen-presenting CD11b^+^ DCs. The data are presented as the mean ± s.e.m. (*n* = 6). Statistical analysis was performed by one-way ANOVA with Fisher’s LSD post-test.


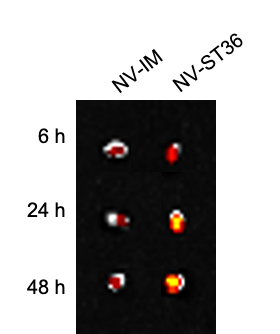


**Fig. 5** Representative IVIS images of dLNs from mice 6, 24, or 48 h post-immunization with RhB-labeled NVs


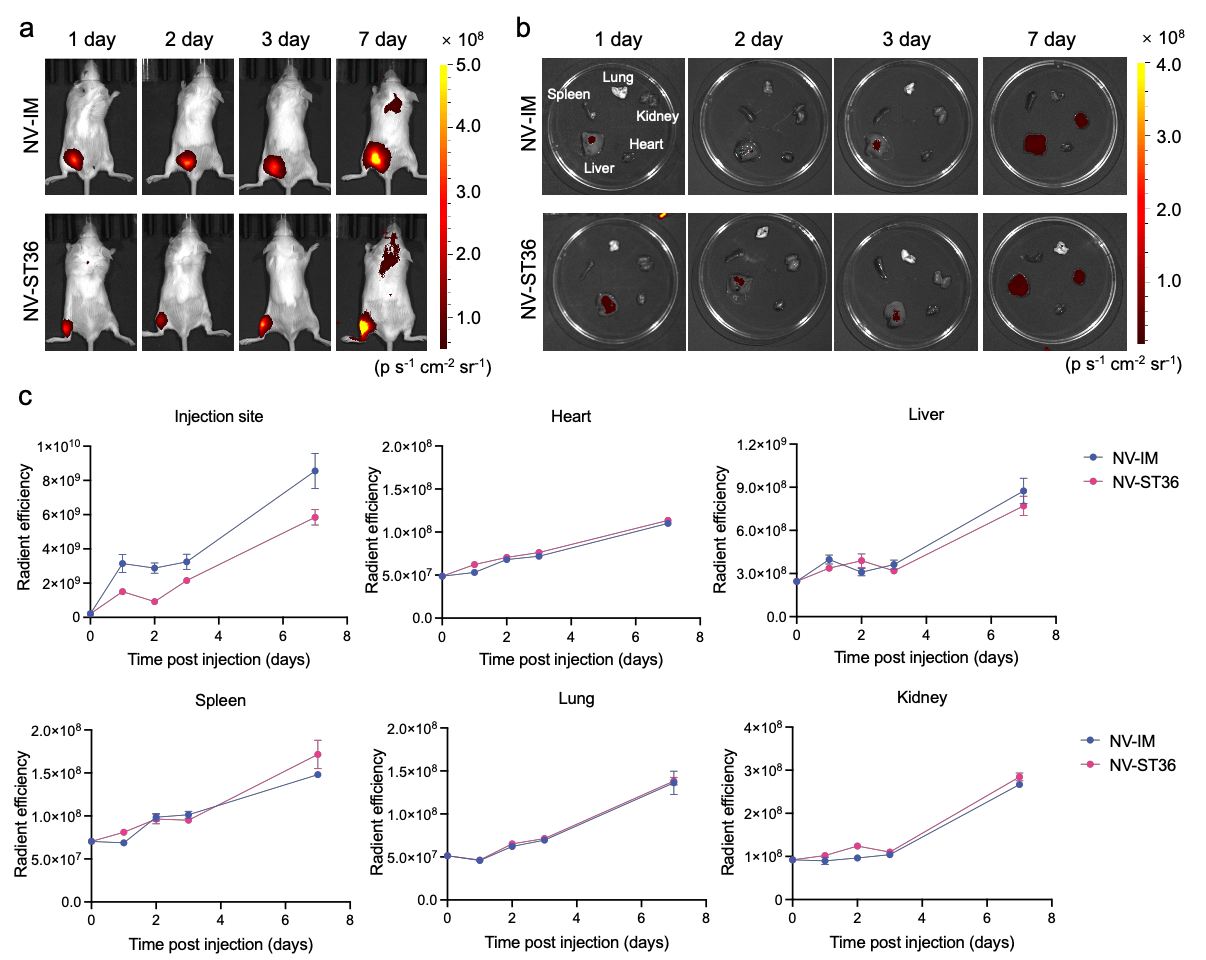


**Fig. 6** **a**, **b**, Representative IVIS images of (**a**) mice and (**b**) sampled organs at 1, 2, 3, or 7 days after injection with Cy5.5-labeled NVs through IM or ST36 route. **c**, Fluorescence intensity derived from the IVIS images of mice or sampled organs. The data are presented as the mean ± s.e.m (*n* = 5)


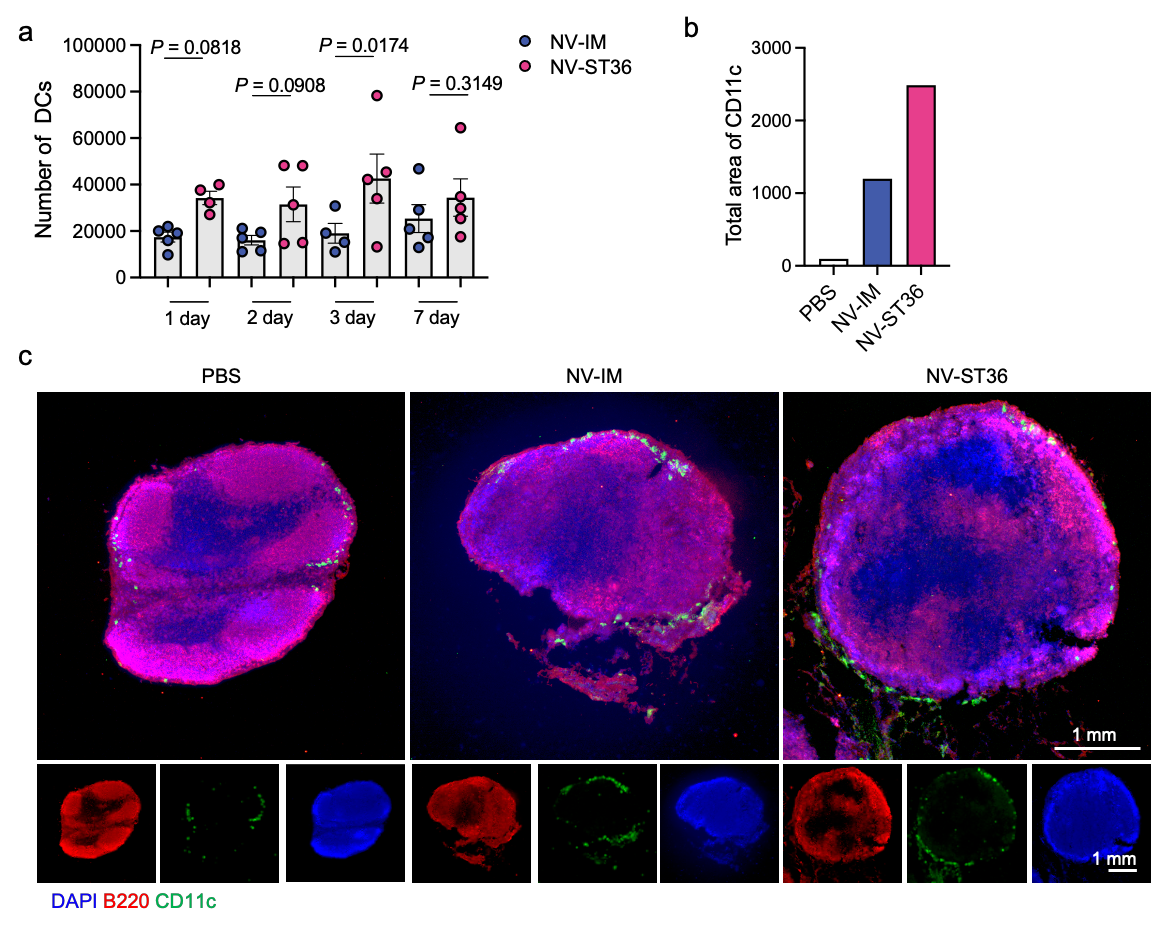


**Fig. 7** **a**, Numbers of DCs in dLNs from mice at 1, 2, 3 and 7 days after injection with NVs through IM or ST36 route. **b**, Quantification of total area of green channel in **a** by Image J. **c**, Representative images of dLN sections stained with anti-B220 antibody (red), anti-CD11c antibody (green), and DAPI (blue) at day 3 post-immunization. The data are presented as the mean ± s.e.m (*n* = 5). Statistical analysis was performed by one-way ANOVA with Fisher’s LSD post-test.


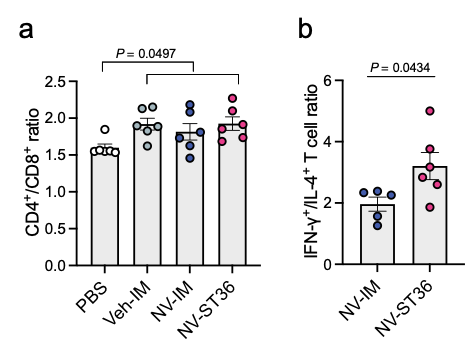


**Fig. 8 a**, Ratio of CD4^+^ T cells to CD8^+^ T cells in the dLNs. **b**, Ratio of IFN-γ^+^ T cells to IL-4^+^ T cells in the dLNs. The data are presented as the mean ± s.e.m (*n* = 6). Statistical analysis in **a** was performed by one-way ANOVA with Fisher’s LSD post-test. Statistical analysis in **b** was performed by Student’s *t*-test


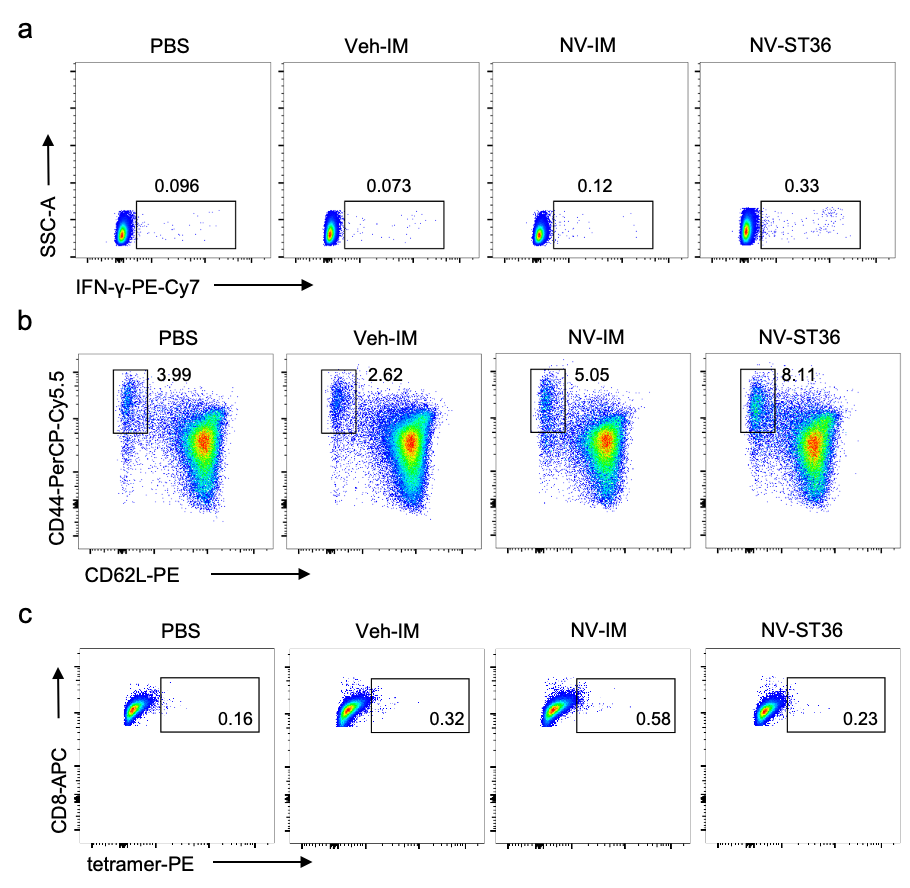


**Fig. 9** Representative flow cytometry scatter plots of (**a**) IFN-γ^+^ T cells, (**b**) Tem cells, and (**c**) tetramer^+^CD8^+^ T cells in dLNs


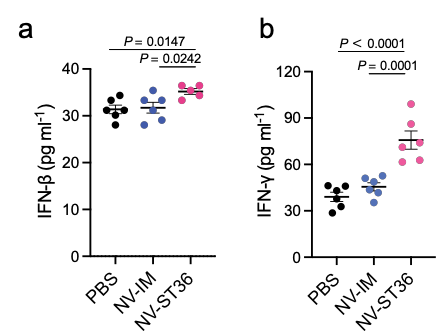


**Fig. 10 a**, **b**, Levels of (**a**) IFN-β and (**b**) IFN-γ in serum measured by ELISA. The data are presented as the mean ± s.e.m (*n* = 6). Statistical analysis was performed by one-way ANOVA with Fisher’s LSD post-test.


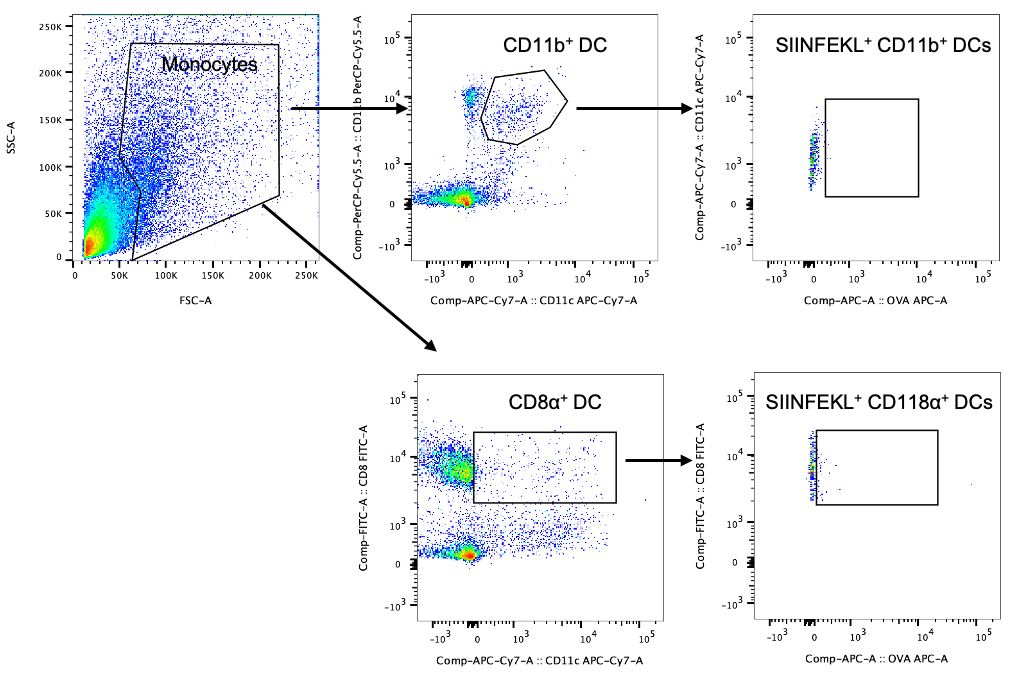


**Fig. 11** Gating strategies to identify SIINFEKL^+^ antigen-presenting CD11b^+^ DCs and CD8α^+^ DCs in dLNs


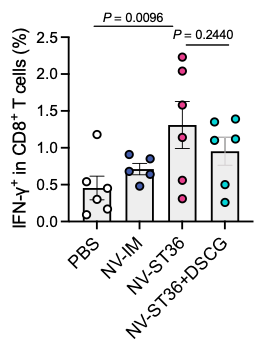


**Fig. 12** Mice were injected with PBS or NVs through IM or ST36 route (*n* = 6). Mice immunized through ST36 additionally received intraperitoneal injection with PBS or DSCG. The dLNs were collected at 24 h post-immunization to detect immune responses. Percentage of IFN-γ^+^ in CD8^+^ T cells in dLNs. The data are presented as the mean ± s.e.m (*n* = 6). Statistical analysis was performed by one-way ANOVA with Fisher’s LSD post-test.


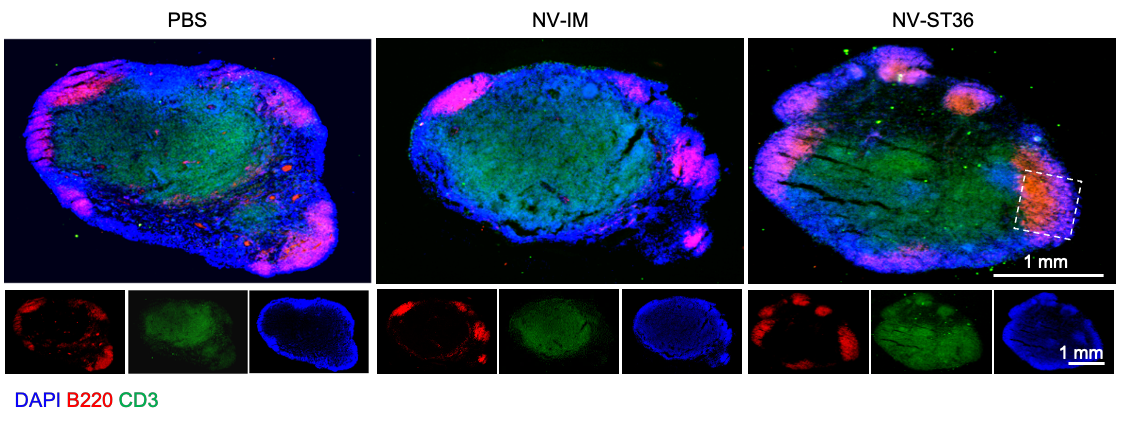


**Fig. 13** Representative images of dLN sections stained with anti-B220 antibody (red), anti-CD3 antibody (green), and DAPI (blue) at 2 days post-immunization. White dotted square represents the enlarged images showing in **Fig. 5f**


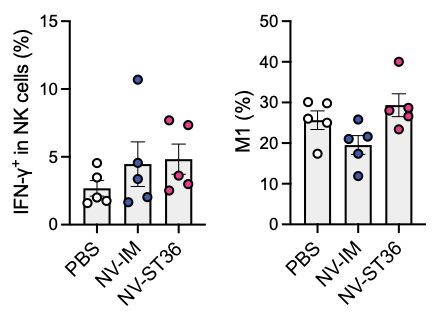


**Fig. 14** Mice were injected with NVs through IM or ST36 route and were euthanatized 72 h post injection for sampling. Percentages of IFN-γ^+^ NK cells (CD3^-^CD49b^+^) and M1 cells (CD86^hi^CD206^low^F4/80^+^CD11b^+^) in dLNs. The data are presented as the mean ± s.e.m (*n* = 5)


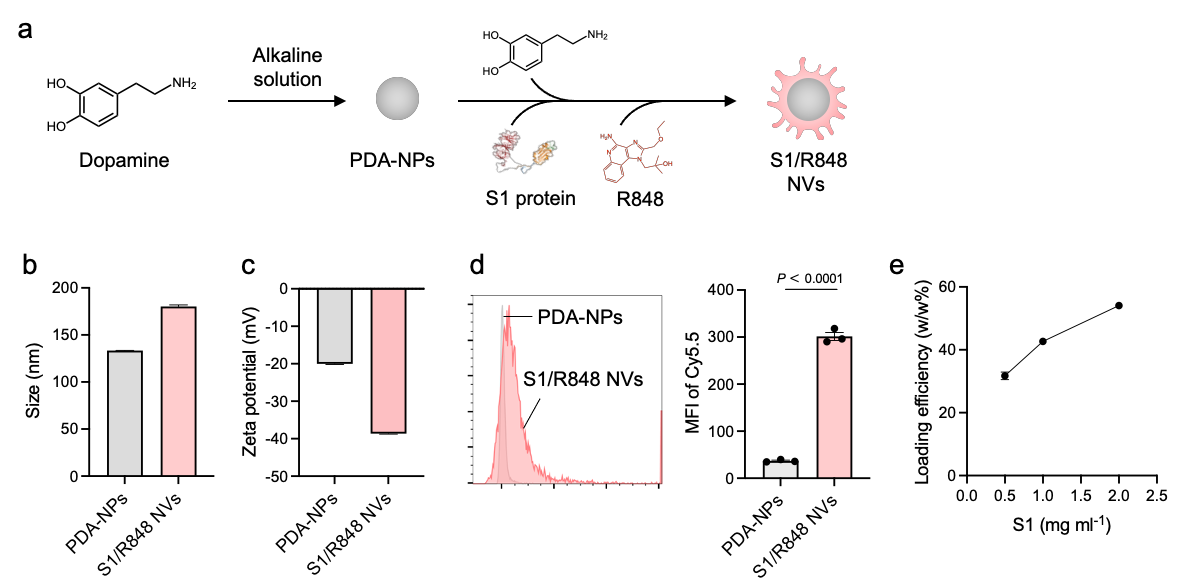


**Fig. 15 a**, Schematic diagram of synthetic route of S1/R848 NVs. **b**,**c**, DLS measurements of (**b**) size distribution and (**c**) zeta potential of S1/R848 NVs. **d**, Flow cytometry histograms and statistic result of PDA-NPs and S1-Cy5.5/R848 NVs. **e**, Loading efficiencies of S1-FITC in NVs at different feed concentrations measured by fluorescence microplate assay. The data are presented as the mean ± s.e.m. (*n* = 3). Statistical analysis was performed using Student’s *t*-test.


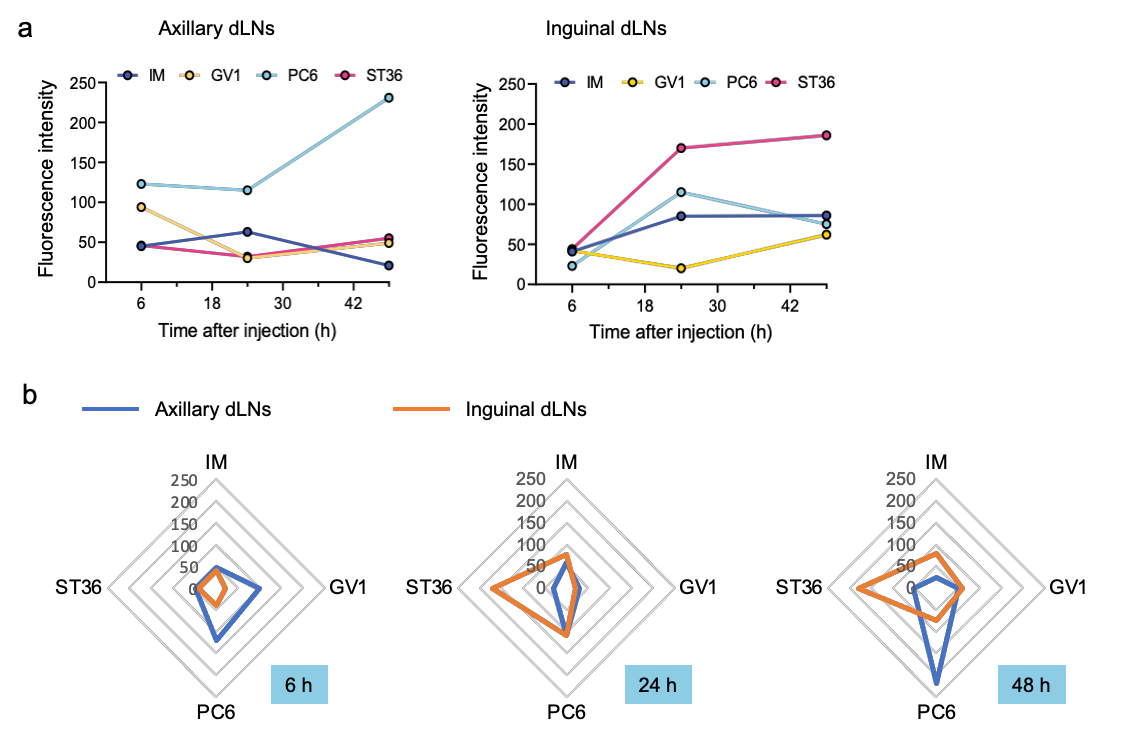


**Fig. 16** **a**, Quantification of fluorescence signals in the axillary and inguinal dLNs of immunized mice through IVIS. **b**, Radar graphs of the correlations between injection sites and dLNs


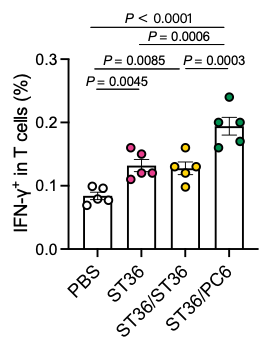


**Fig. 17** Percentages of IFN-γ^+^ in T cells in dLNs from mice treated with combination-route ADMI, respectively. The data are presented as the mean ± s.e.m. (*n* = 5). Statistical analysis was performed by one-way ANOVA with Fisher’s LSD post-test


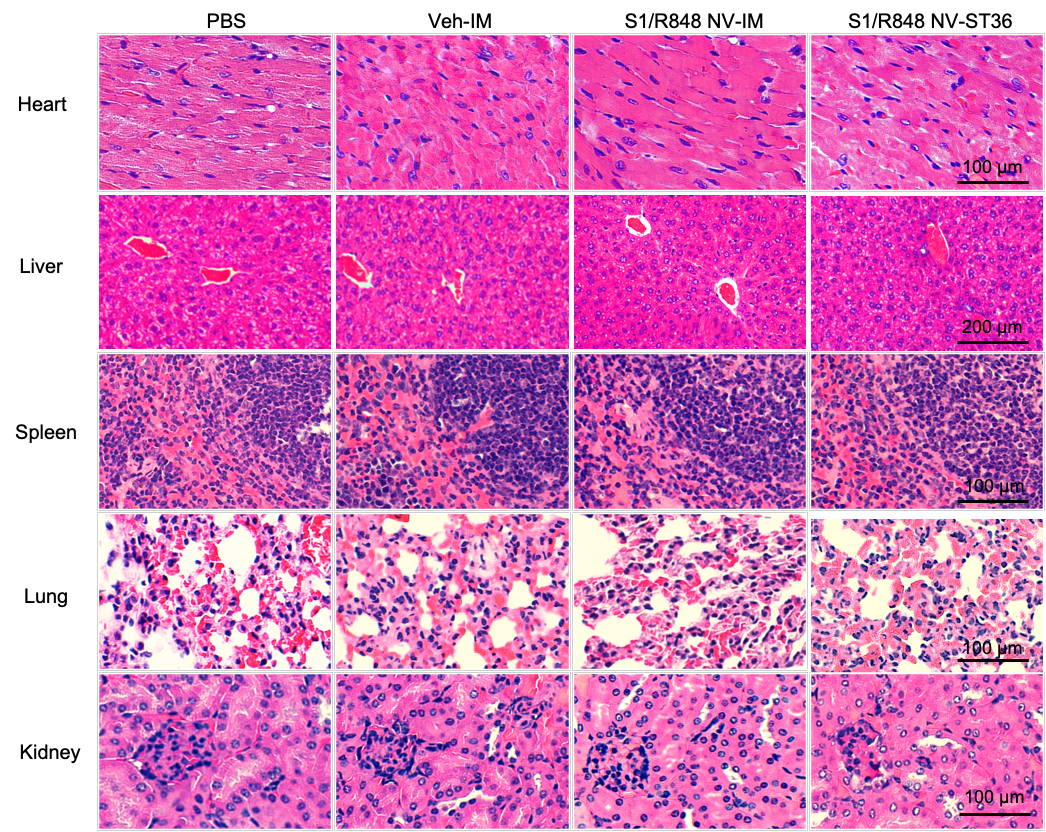


**Fig. 18** Representative H&E staining images of the heart, liver, spleen, lung, and kidney, respectively. Mice were immunized with PBS, vehicle, or S1/R848 NVs by IM or ST36 route on day 0 and 21 and euthanized on day 35 for tissues collection


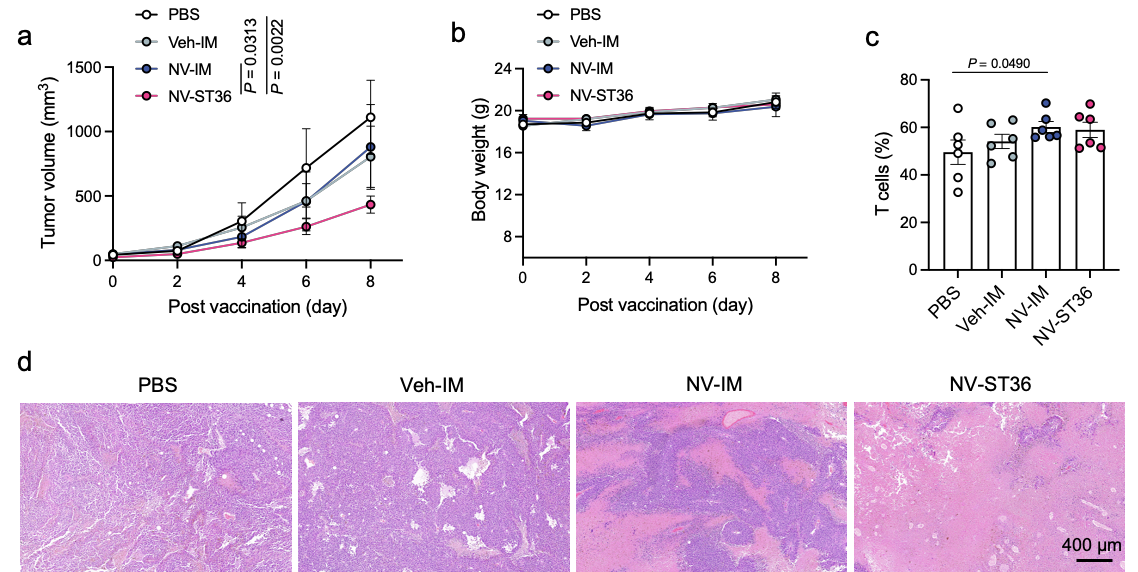


**Fig. 19** **a**, Tumor growth monitored over time. **b**, Variation of body weight from treated mice. **c**, Percentage of T cells in dLNs from treated mice measured by flow cytometric analysis. **d**, Representative H&E staining images of tumor tissues from treated mice. The data are presented as the mean ± s.e.m. (*n* = 6). Statistical analysis in **a** was performed by two-way ANOVA with the Bonferroni correction post-test. Statistical analysis in **c** was performed by one-way ANOVA with Fisher’s LSD post-test


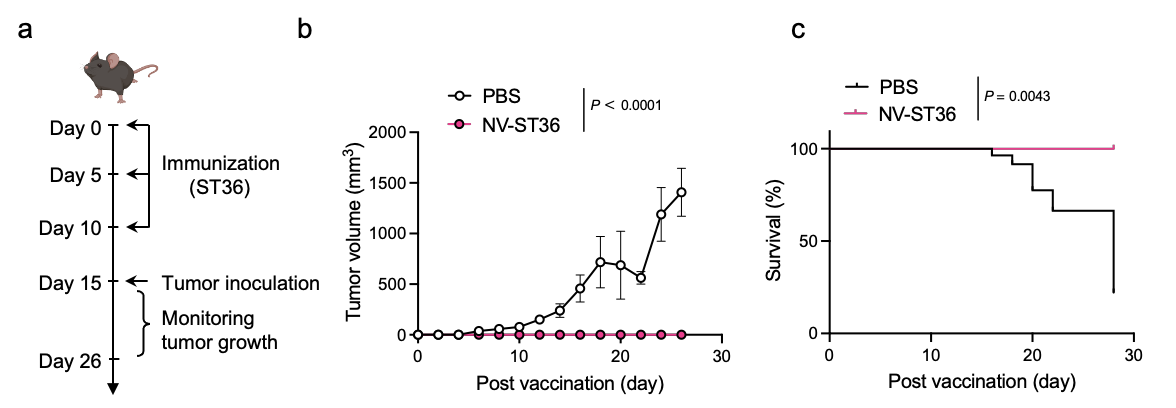


**Fig. 20 a**, Experimental design of evaluating ADMI-induced protective effectiveness in prophylactic model. C57BL/6 mice (*n* = 6 per group) were injected with NVs on day 0, 5, 10 by ST36 route. On day 15, immunized mice were challenged with B16-OVA cells. **b**, Monitoring of tumor growth after different treatments over time. **c**, Survival curves of mice after different treatments. Statistical analysis among tumor sizes in **b** was tested using a two-way ANOVA with the Bonferroni correction post-test. Statistical analysis between survival curves was performed by log-rank (Mantel-Cox) test
